# Comprehensive Genome‐Wide Identification and Expression Analysis of the N6‐Methyladenosine (m6A) Regulatory Network Influences Rapid Stress Adaptation With Exogenous Melatonin in Rice

**DOI:** 10.1111/jpi.70109

**Published:** 2026-01-13

**Authors:** Hira Khanzada, Ghulam Mustafa Wassan, Ping Wang, Saba Khanzada, Xiaoning Wang, Zhihui Xia

**Affiliations:** ^1^ Sanya National Center of Technology Innovation for Saline‐Alkali Tolerant Rice, School of Breeding and Multiplication (Sanya Institute of Breeding and Multiplication), Hainan University Sanya Hainan China; ^2^ Hainan Key Laboratory of Crop Genetics and Breeding, Institute of Food Crops, Hainan Academy of Agricultural Sciences Haikou Hainan China; ^3^ Hira Mustafa Crop Sciences, HM Seed Karachi Sindh Pakistan; ^4^ Department of Plant Breeding and Genetics Faculty of Crop Production, Sindh Agriculture University Tandojam Sindh Pakistan

**Keywords:** Cis‐regulatory elements, exogenous melatonin, expression profile, N6‐methyladenosine (m6A), RNA methylation, secondary metabolite biosynthesis, stress‐responsive genes

## Abstract

Exogenous melatonin has emerged as a pivotal multifunctional signaling molecule, recognized for its critical role in enhancing stress tolerance and improving crop productivity. N6‐methyladenosine (m6A) is the most prevalent internal modification found in eukaryotic mRNA and plays a crucial role in regulating plant growth, development, and stress responses. Despite its importance, the regulatory mechanisms of the m6A pathway in rice exposed to exogenous melatonin remain inadequately investigated. This study investigates systematic analysis of m6A‐regulatory gene families in rice. We identified a total of 124 genes, which include 7 writers, 22 readers, and 95 erasers. The distribution of these genes is uneven across the 11 chromosomes of the rice genome. Analysis of conserved domains revealed structural signatures that are specific to each gene family. Phylogenetic relationships with dicot and monocot species offered insights into evolutionary trajectories. Notably, gene structure and motif analyses revealed functional divergence within and between gene families. Cis‐element analysis identified abundance motifs associated with stress adaptation, hormonal signaling, and TFs, including ABRE, DRE, and MYB. Furthermore, synteny analysis unveiled both conserved regions and lineage‐specific expansions, particularly within the YTH and ALKBH families. The protein interaction network revealed robust connections among subgroups and identified 10 hub genes. GO and KEGG analyses indicated significant enrichment in stress‐related pathways, including secondary metabolite biosynthesis, flavonoid biosynthesis, and cysteine and methionine metabolism. RT‐qPCR validates that melatonin Osm6As significantly influences the expression of targeted genes, with melatonin upregulations exhibiting a time‐dependent pattern. Furthermore, GFP tagging of OsECT2 revealed that protoplasts are evenly distributed, suggesting robust nuclear enrichment of fluorescence. This study offers new insights into the epitranscriptomic regulatory responses of the m6A‐modifier.

## 1 Introduction

Epigenetic modifications of RNA have recently garnered recognition as critical modulators of post‐transcriptional gene regulation. Specifically, epitranscriptomics has emerged as a dynamic and expanding area of biological research that contributes to the regulation of gene expression [1, 2]. To date, over 170 distinct RNA modifications have been reported in various types of RNA, encompassing long noncoding RNA (lncRNA), microRNA (miRNA), messenger RNA (mRNA), ribosomal RNA (rRNA), and transfer RNA (tRNA). It is noteworthy that the RNA alterations that constitute the “epitranscriptome” play an essential role in regulating gene expression and various molecular and cellular processes, including plant adaptation to stress resilience and signaling [3, 4]. Among these modifications, N6‐methyladenosine (m6A) is the most abundant internal modification identified in eukaryotic mRNA. Gene expressions are regulated by multifunctional processes that occur at both transcriptional and post‐transcriptional levels [5]. These regulatory mechanisms are critical throughout plant developmental processes and stress adaptation. The m6A represents a novel layer of post‐transcriptional gene regulation. The presence of the m6A mark on mRNA can influence its abundance and localization, as well as its potential for splicing. This mechanism is critically linked to the expression of essential proteins [6, 7]. Although the biological mechanisms in plants remain poorly understood compared with those of humans and animals.

The genetic and molecular investigations have shown that the m6A modification, recognized by m6A‐binding proteins commonly referred to as “writers,” adds a methyl group, and “readers,” which bind to m6A sites, determine the fate of RNA [2]. These processes are essential for regulating RNA processing and metabolism, influencing RNA stability, translation efficiency, and degradation. Furthermore, m6A “erasers” specifically demethylases, modulate the abundance of m6A modifications, and play critical roles in their alteration and distribution [8]. Among them, the readers play a more specific regulatory role by binding to m6A modification sites on RNA [9]. Readers initially discovered in mammalian cells predominantly comprise a conserved YTH (YT521‐B homology) domain, which includes two human subtypes, YTHDF and YTHDC [10]. The YTH domain, which is conserved across both animals and plants, contains crucial tryptophan residues that are essential for m6A binding. This binding facilitates the biological functions associated with mRNA methylation [5, 11, 12]. In plants, the YTH‐domain proteins were initially classified as members of the ECT family, which share conserved C‐terminal regions and regulate mRNA fate [6, 13]. Recent investigations have elucidated the critical roles of specific m6A pathway genes, particularly reader genes, in plant biology. For instance, the Arabidopsis ECT2 and ECT3 genes are essential for regulating both the formation and timing of leaf morphogenesis [13]. Collectively, these studies underscore the critical importance of m6A modifications in mRNA for plant developmental processes. However, the biological function and the behavior of individual m6A in crops such as rice remain rarely characterized [14].

Recent studies have demonstrated that RNA modifications, pivotal components of the epitranscriptome, play a critical role in regulating gene expression and have the potential to adapt swiftly to plant growth, development, and responses to environmental stress [15, 16]. The transgenic Arabidopsis plants overexpressing ECT8 show enhanced salt tolerance, attributed to increased stability and expression levels, which are reported as a positive regulator in response to salt stress [17]. Additionally, Arabidopsis exhibits dynamic changes in m6A levels following a 6‐h treatment with 150 mM NaCl [18]. The m6A writer in Arabidopsis has been shown to positively regulate photosynthesis by enhancing m6A modifications in the transcripts of genes associated with photoprotection, thereby improving their stability and translational efficiency [19]. Although previous research provides substantial evidence that the mechanism by which the m6A modifier responds to environmental stresses in plants remains unclear.

Melatonin (N‐acetyl‐5‐methoxytryptamine, MT) is a crucial biological hormone that plays a distinct role in biological function [20]. In plants, MT functions as an antioxidant, signaling molecule, and regulator of stress adaptation pathways. Melatonin mitigates growth inhibition by improving Na^+^/K^+^ homeostasis and has been reported in several plant species, such as tomato [21], Cucumber [22], Basil (*Ocimum basilicum* L.) [23], and Rice [24]. Exogenous melatonin influences transcriptional and post‐transcriptional activity during stress, suggesting a possible interaction with RNA regulatory pathways, including m6A [25]. Melatonin association priming enhances abiotic stress, including heat, cold, drought, and salt, by regulating genes involved in cis elements such as DRE, HSF, SOS, and ABA pathways. The application of melatonin induced profound changes in gene expression, including transcription factor (TFs) ERF, MYB, NAC, and WRKY [26]. Moreover, miRNAs, including DREB, MYB, bHLH, CDPK, and WRKY, have been reported to enhance expression in response to melatonin application [27]. However, relatively little is known about its insight target genes and regulatory network. Furthermore, the molecular mechanism underlying exogenous melatonin signaling in regulating epitranscriptomic modification, specifically m6A, remains largely unknown in crops. The purpose of this study is to conduct a systematic analysis of the m6A modifier, including writer, reader, eraser, and its role under the effect of melatonin, which could provide valuable insights for future studies in plants.

## 2 Materials and Methods

### 2.1 Sequencing, Retrievhal, and Gene Identification of the m6A Modifier: Writer, Readers, and Erasers

The m6A modifier protein sequences of *Arabidopsis thaliana* were retrieved from NCBI (https://www.ncbi.nlm.nih.gov/protein) and utilized as reference query sequences to the *Oryza sativa* genome to obtain corresponding m6A protein sequences. The genomic sequence data, along with annotation files for *Oryza sativa*, were retrieved from the Rice Genome Annotation Project database (https://rice.uga.edu/index.shtml) [28]. All predicted m6A sequences were retrieved using BLASTP, with an E‐value cutoff of 1 × 10^5^, to minimize false positives. We used HMMER search with an E‐value threshold of < 1 × 10^5^. Only sequences satisfying both criteria were retained for further analysis. Subsequently, the Pfam database was used to validate the Writer (PF05063), Reader (PF04146), and Erasers (PF03171) domains (http://pfam.xfam.org). Furthermore, the identified Osm6As proteins were confirmed by examining their conserved domains within their respective gene families. The CDD database (https://www.ncbi.nlm.nih.gov/Structure/cdd/cdd.shtml) was utilized to determine the presence of conserved structural domains in m6A modifier proteins [29]. Additionally, all identified m6As proteins underwent further scrutiny and validation using the SMART database (http://smart.emblheidelberg.de/). Finally, any duplicate or redundant protein with a similar sequence length was manually reviewed and excluded from subsequent analysis.

### 2.2 In Silico Subcellular Localization and Examination of Protein Characteristics

To elucidate the physicochemical properties of the Osm6A modifier, assessments were conducted using the Protparam tool provided by ExPASY (https://web.expasy.org/protparam/) [30]. This analysis encompassed various parameters, including the number of amino acids, chromosome length, molecular weight (MW), isoelectric points (pI), and the grand average of hydropathicity (GRAVY). To predict the subcellular localization of Osm6A proteins, the CELLO (https://cello.life.nctu.edu.tw/) database was utilized [31].

### 2.3 Conserved Motifs, Conserved Domains, and Gene Structure Analysis of Osm6As

To identify the motifs within each m6A‐related gene family, the MEME website (https://meme‐suite.org/meme/tools/ame) was utilized with the number of motifs set to 10, and other parameters were maintained at default settings. Subsequently, the structural characteristics of m6A‐related genes were investigated by analyzing conserved motifs and intron distribution. The genomic location information for each m6A modifier subgroup, including General Feature Format Version 3 (GFF3) data for rice (*Oryza sativa*), was input into the Gene Structure View (Advanced) program of TBtools‐II software to visualize their phylogenetic tree, motif composition, conserved domain, and introns‐exons structures [32].

### 2.4 Phylogenetic Tree Construction of m6A‐Related Proteins

To gain insights into the evaluation of m6A modifier proteins, these proteins were classified into three functional categories: writers, readers, and erasers. Subsequently, the genomes of *Arabidopsis thaliana*, *Triticum aestivum*, *Zea mays*, *Sorghum bicolor*, and *Glycine max* were utilized for phylogenetic analysis, and genomic data were obtained from the Ensembl Plants database (https://plants.ensembl.org/index.html). The Blast Zone plugin in TBtools‐II was used to perform BLAST searches to obtain homologous sequences from monocot and dicot plant species. Furthermore, MEGA 12 software was used to align the protein sequences for each category (writers, readers, and erasers). Subsequently, phylogenetic trees for the m6A‐related protein families were constructed for each category using the Maximum Likelihood Method (MLM) in MEGA 12, with 1000 bootstrap replicates. Lastly, the phylogenetic trees for each category of m6A‐related modifiers were illustrated using the iTOL website (iTOL: Interactive Tree Of Life) [33].

### 2.5 Mining of Cis‐Acting Elements of Osm6As Modifier

Understanding the mechanisms of gene expression regulation, particularly those that depend on cis‐regulatory elements, is essential. To identify these regulatory elements in the promoter sequences of Osm6A genes, 2000 bp upstream regions were retrieved. Subsequently, these sequences were analyzed for cis‐acting elements and uploaded to the PlantCARE website (https://bioinformatics.psb.ugent.be/webtools/plantcare/html/) [34]. The identified cis‐elements were then categorized, statistically analyzed, and visualized using the Basic BioSequence View program in TBtools‐II software [32].

### 2.6 Collinearity Analysis and Chromosome Location

The localization of all three categories of Osm6A genes was determined from their genomic positions, as retrieved from the *Oryza sativa* GFF3 file. Chromosome mapping was performed and visualized using TBtools‐II [32]. To analyze gene duplication events within the gene family based on copy numbers and genomic distributions, the Multiple Collinearity Scan toolkit (MCScanX) program in TBtools‐II, which is effective for classifying the origins of gene duplication, was employed to investigate the duplication events of m6A modifier genes within *Oryza sativa* and their collinear relationships with *Arabidopsis thaliana, Sorghum bicolor, and Glycine max*. Genome sequences and annotation data files were retrieved from the Ensembl Plants database (https://plants.ensembl.org/index.html). Subsequently, collinearity analysis was conducted using the Multiple Synteny Plot program in TBtools‐II [32].

### 2.7 Expression Profile Analysis of Osm6As Genes Determined by Transcriptome Sequences

To elucidate the expression profile of m6A‐related genes in *Oryza sativa*, RNA‐seq data were retrieved from the Rice Genome Annotation Project Database (http://rice.uga.edu), which hosts a curated collection of mRNA‐seq libraries from the NCBI Sequence Read Archive (SRA). The selected datasets comprised 12 distinct tissue samples (stem, seedling, leaf, leaf‐flag, root, anther, panicle, embryo, inflorescence, seed, pistil, and seeds at 5 DPA). The SRA numbers for the datasets are listed in Supporting Information: Table S1. Additionally, RNA‐seq data for various abiotic stress conditions, including drought, salt, cold, heat, germination imbibition, and mature seed hypoxia, along with their corresponding controls (CK), were retrieved from the same repository. The corresponding SRA accession numbers for stress datasets used in this study are provided in Supporting Information: Table S2. The raw sequences were aligned to the *Oryza sativa* reference genome using kallisto (version 0.51). Gene expressions were quantified as Transcripts Per Kilobase Million (TPM) for each biological replicate, and TPM values were log2‐transformed to stabilize variance. A substantial amount of data underwent quality control filtering, including the exclusion of genes with TPM < 1 across all replicates. The gene or locus name was used to search for and retrieve TPM data. For multiple sequence libraries, the average TPM datasets were visualized as cluster heatmaps using Tbtools‐II [32].

### 2.8 Protein–Protein Interactions (PPI) Network and Functional Enrichment Analysis

To understand the complex nature of proteins and their interactions, which are crucial for various biological functions, it is imperative to analyze their interconnected networks. Such an analysis requires a careful investigation of these relationships [35]. In order to investigate functional associations among m6A‐related proteins in rice, a comprehensive PPI network and subsequent functional enrichment analysis were conducted using the STRING database (https://cn.string‐db.org) [36]. The STRING database provides a thorough and objective global network of PPI predictions, along with their associated relationships [36]. All m6A‐related machinery genes were submitted to the STRING database to construct the PPI network, with the maximum number of interactions set to 20 for the first shell and 5 for the second shell. In the PPI network analysis, proteins were represented as vertices and their interactions as edges. To identify functional modules within the network, the Markov clustering (MCL) algorithm was employed with default parameters. This method simulates stochastic flow on the interaction graph and groups proteins according to interaction density. Consequently, three distinct clusters emerged, broadly corresponding to the m6A regulatory categories as writers, readers, and erasers. The PPI network was subsequently exported and further analyzed using Cytoscape [37]. To analyze the network attributes of each node, the Cytoscape plugin cytoHubba was utilized to identify hub nodes, with the top 10 nodes defined as hub based on degree [38]. The database STRING also attempts to assign the submitted proteins to their corresponding pathways and functional enrichments [35], across three Gene Ontology categories: biological process (BP), cellular component (CC), and molecular function (MF) [39], as well as KEGG pathways [40]. Subsequently, all 124 Osm6A genes from the three subgroups (writer, reader, and eraser) were subjected to functional enrichment analysis via GO and KEGG.

### 2.9 Plant Material and Growth Conditions

To investigate the response of Osm6A gene expression in response to various stresses, a total of 10 groups of rice plants were involved, each group had three replications including groups were used as control (CK), drought treated plants, salt treated plants, cold treated plants, heat treated plants, exogenous melatonin (MT) treated plants as well as phytohormonal such as jasmonic acid (JA), salicylic acid (SA), and gibberellic acid (GA) treated plants. The rice cultivar seed (TP309) was selected and then subjected to disinfection. A total of 0.01% HgCl_2_ for 6 min. Subsequently, the sterilized seed was washed several times with distilled water. Seeds were incubated in the dark at 28°C for 4 days to allow germination. The seeds were allowed to grow for 1 week in Yoshida nutrition solution as previously described [41, 42]. Following 2‐week growth periods, seedlings were subjected to specific treatments for drought, 20% PEG (polyethylene 6000), salinity (150 mM NaCl), cold 4°C, heat 45°C. The drought and salt‐stress‐exposed plants were treated with melatonin MT 100 μM, on alternate days [43], and JA 100 μM, SA 100 μM, and GA 100 μM were used as described by Yimer, Nahar, Kyndt, Haeck, Meulebroek [44]. Subsequently, water was utilized as a control (CK). For RNA extraction, fully expanded uppermost leaves were collected at multiple time points, such as 0 (CK), 12, 24, and 48 h after treatment.

### 2.10 Vector Construction

The full‐length coding sequence (CDS) without the stop codon of OsECT2 was amplified and inserted into the pCAMBIA1300‐GFP vector using the fusion GFP at the C‐terminal of OsECT2 under the CaMV 35S promoters to construct 35::OsECT2‐GFP. Subsequently, the empty vector 35::GFP served as a control expression.

### 2.11 Rice Protoplasts Isolation

To investigate OsECT2 expression, 10‐day‐old rice seedlings were used to develop protoplasts. The seedling was used by removing the outer leaf sheath, and the inner elongation were cut into small pieces about 0.5 mm and incubated in enzyme solution (cellulase + macerozyme) for 4 h, followed by centrifuging at 80–100 rpm under dark conditions for enzymatic hydrolysis for 5 h. Subsequently, the digested material was filtered through a 40 µm nylon gauze by keeping the filtrate in the middle of a 2 mL EP tube and gently squeezing the tube. The protoplast was obtained by centrifuging at 600 rpm for 5 min at 4°C. The pellet was rinsed twice with 1 mL of precooled W5 solution, then resuspended in W5, and incubated for 30 min on ice.

### 2.12 Transient Expression in Protoplast

The protoplasts were centrifuged at 600 rpm for 5 min after settling, and the supernatant was resuspended in MMG solution. Subsequently, for each transformation, 50 μL of protoplast solution and 10 μL of DNA plasmid were used. The mixture was incubated for 8–10 min before examination under a microscope. Consequently, 20–40 protoplasts per field of view at ×40 magnification with a plasmid ratio of 10 μL of the OsECT2 gene plasmid, 50 μL of protoplasts, and an equal volume of 60 μL protoplasts with 40% PEG4000 solution. To examine the expression pattern, 60 μL of protoplast mixture with 40% PEG4000 solution was slowly inverted and mixed after incubation in a water bath at 24.5°C for 15–20 min.

### 2.13 Green Fluorescence Protein (GFP) Confocal Microscopy

The expression of protoplasts for 35S::OsECT2‐GFP and 35S::GFP was examined using a laser scanning confocal microscope (×100 oil objective). The excitation wavelength of 488 nm was used to determine GFP. Subsequently, GFP images were acquired for the GFP channel, the bright‐field, and the merged composite. Localization was assessed by comparing nuclear versus cytoplasmic GFP distribution.

### 2.14 RNA Extraction and q‐RT PCR Expression Analysis

Total RNA was extracted utilizing the TRIzol reagent (Invitrogen, Carlsbad, CA, USA) and subsequently purified with the RNeasy Plant Mini Kit (Qiagen, CA, USA) according to the manufacturer's instructions. The RT‐qPCR was performed on the SYBR Green I (Osaka, Japan), following the reaction conditions [45]. The gene‐specific primers for each Osm6As were designed by utilizing the NCBI Primer‐Blast program (Supporting Information: Table S3). For each treated sample, three independent replications were conducted. The Rice *Actin1* gene was used as an internal control [46, 47]. The comparative cycle threshold (Ct) method 2^−ΔΔCT^ was employed to quantify relative expression levels [45].

### 2.15 Statistical Analysis

The statistical analysis was performed using SPSS software (V. 9.1, SPSS Institute, Inc. NC, USA). The mean ± standard deviation (SD) was derived from three biological replicates. Statistical significance relative to the control (CK) was determined using a two‐tailed Student's *t*‐test, with asterisks denoting levels of significance *p* < 0.05 (*), *p* < 0.01 (**), and *p* < 0.001 (***), while “ns” indicates no significant difference.

## 3 Results

### 3.1 Identification and Characterization of m6A‐Regulator Genes

Homologous proteins are defined by their analogous amino acid sequences, which often serve similar functional roles across different species or within the same species. These proteins consist of functional units, known as domains, which are frequently linked to diverse functions [48]. A total of 124 Osm6A genes were identified, comprising 7 writers, 22 readers, and 95 erasers. The physicochemical properties and in silico subcellular localization of the Osm6A genes were analyzed and summarized in Table 1. The Osm6A writer proteins ranged from 427 to 1013 amino acids (aa) in length, with MWs ranging from 0.85 to 113.61 kDa and theoretical pI values ranging from 6.17 to 8.49. While in the readers, the protein ranged from 406 to 709 aa in length, with MW ranging from 44.06 to 78.20 kDa, the theoretical pI values ranged from 5.13 to 9.11. The eraser protein ranged from 236 to 544 aa in length, with MWs of 26.59 to 59.35 kDa, and theoretical pI values ranging from 4.75 to 9.37. Furthermore, in silico analysis of subcellular localization revealed that all writer and reader genes are primarily found in the nucleus. Moreover, the eraser genes were predominantly localized in the cytoplasm, chloroplasts, mitochondria, nucleus, and the plasma membrane. The Grand Average of Hydropathicity (GRAVY) values for all Osm6A proteins, except OsMTA‐7, demonstrated hydrophobic characteristics, as evidenced by positive values. In contrast, the majority of Osm6A proteins were predominantly hydrophilic.

**Table 1:** Characteristic summary of the identified m6A‐related genes in rice *Oryza sativa*.

Group	Transcript ID	Gene name	Chr ID start‐end stand	Protein length (A.A)	MW (kDa)	pI	GRAVY	Subcellular localization
Writers	LOC_Os01g16180.1	OsMTA‐1	Chr1_9159606‐9165693 +	764	0.85	6.75	−0.94	Nuclear
Writer	LOC_Os01g16180.3	OsMTA‐2	Chr1_9159606‐9165693 +	753	83.91	6.73	−0.956	Nuclear
Writer	LOC_Os02g45110.1	OsMTA‐3	Chr2_27360417‐27365800 +	706	77.80	6.76	−0.456	Nuclear
Writer	LOC_Os03g05420.1	OsMTA‐4	Chr3_2676953‐2682146 −	753	83.59	6.75	−0.934	Nuclear
Writer	LOC_Os03g05420.2	OsMTA‐5	Chr3_2678302‐2682168 −	677	74.94	6.33	−0.881	Nuclear
Writer	LOC_Os03g10224.1	OsMTA‐6	Chr3_5202705‐5206440 −	427	49.39	8.49	−0.55	Nuclear
Writer	LOC_Os10g31030.1	OsMTA‐7	Chr10_16201250‐16208251 +	1013	113.61	6.17	1.188	Nuclear
Readers	LOC_Os01g22630.1	OsECT‐1	Chr1_12725141‐12731526 +	708	78.20	8.68	−0.564	Nuclear
Readers	LOC_Os01g48790.1	OsECT‐2	Chr1_27983688‐27990383 +	609	67.07	5.650	−0.661	Nuclear
Readers	LOC_Os01g48790.2	OsECT‐3	Chr1_27984772‐27990383 +	598	65.76	5.570	−0.629	Nuclear
Readers	LOC_Os01g48790.3	OsECT‐4	Chr1_27985319‐27990383 +	458	50.78	7.610	−0.698	Nuclear
Readers	LOC_Os03g06240.1	OsECT‐5	Chr3_3125933‐3130483 −	708	76.57	8.100	−0.693	Nuclear
Readers	LOC_Os03g06240.2	OsECT‐6	Chr3_3125933‐3130483 −	707	76.45	8.100	−0.689	Nuclear
Readers	LOC_Os03g20180.1	OsECT‐7	Chr3_11402649‐11408108 +	709	77.05	5.500	−0.657	Nuclear
Readers	LOC_Os03g53670.1	OsECT‐8	Chr3_30777720‐30781832 +	661	71.62	8.350	−0.796	Nuclear
Readers	LOC_Os03g53670.2	OsECT‐9	Chr3_30777720‐30781832 +	662	71.75	8.350	−0.800	Nuclear
Readers	LOC_Os04g04000.1	OsECT‐10	Chr4_1843006‐1848258 +	675	76.44	6.08	−0.909	Nuclear
Readers	LOC_Os04g51940.1	OsECT‐11	Chr4_30821134‐30825520 +	574	63.79	5.58	−0.697	Nuclear
Readers	LOC_Os04g51940.2	OsECT‐12	Chr4_30821130‐30825520 +	568	63.12	5.47	−0.698	Nuclear
Readers	LOC_Os05g01520.1	OsECT‐13	Chr5_304131‐311132 −	638	73.17	6.9	−0.789	Nuclear
Readers	LOC_Os05g06740.1	OsECT‐14	Chr5_3515604‐3518444 −	406	44.07	9.11	−0.365	Nuclear
Readers	LOC_Os06g46400.1	OsECT‐15	Chr6_28151360‐28156784 +	665	72.60	6.32	−0.958	Nuclear
Readers	LOC_Os07g07490.1	OsECT‐16	Chr7_3726574‐3731170 −	602	66.24	7.61	−0.795	Nuclear
Readers	LOC_Os07g07490.2	OsECT‐17	Chr7_3726574‐3731170 −	578	63.60	7.99	−0.825	Nuclear
Readers	LOC_Os08g12760.1	OsECT‐18	Chr8_7559098‐7563188 −	577	63.87	5.41	−0.789	Nuclear
Readers	LOC_Os08g12760.2	OsECT‐19	Chr8_7559098‐7563188 −	570	63.06	5.47	−0.779	Nuclear
Readers	LOC_Os08g12760.3	OsECT‐20	Chr8_7559098‐7563158 −	562	62.25	5.4	−0.800	Nuclear
Readers	LOC_Os08g12760.4	OsECT‐21	Chr8_7559098‐7563170 −	481	53.47	5.31	−0.778	Nuclear
Readers	LOC_Os08g44200.1	OsECT‐22	Chr8_27825032‐27830316 −	624	68.27	5.13	−0.707	Nuclear
Erasers	LOC_Os01g08220.1	OsALKBH‐1	Chr1_4003659‐4004946 −	373	40.57	6.47	−0.109	Chloroplast
Erasers	LOC_Os01g09300.1	OsALKBH‐2	Chr1_4726659‐4731243 +	361	40.87	5.35	−0.331	Cytoplasmic
Erasers	LOC_Os01g11150.1	OsALKBH‐3	Chr1_5968819‐5972489 −	335	35.14	6.61	−0.071	Cytoplasmic
Erasers	LOC_Os01g22910.1	OsALKBH‐4	Chr1_12875498‐12882540 −	464	49.56	6.03	−0.376	Nuclear
Erasers	LOC_Os01g25010.1	OsALKBH‐5	Chr1_14101994‐14108083 +	350	38.67	5.13	−0.188	Cytoplasmic
Erasers	LOC_Os01g27490.1	OsALKBH‐6	Chr1_15346349‐15347941 −	375	40.71	5.38	−0.195	Cytoplasmic
Erasers	LOC_Os01g35230.1	OsALKBH‐7	Chr1_19505311‐19508412 −	396	41.63	5.91	−0.15	Chloroplast
Erasers	LOC_Os01g39860.1	OsALKBH‐8	Chr1_22489496‐22491483 +	312	34.03	5.21	−0.206	Cytoplasmic
Erasers	LOC_Os01g55240.1	OsALKBH‐9	Chr1_31795105‐31797643 +	327	35.33	6.23	−0.108	Cytoplasmic
Erasers	LOC_Os01g61610.1	OsALKBH‐10	Chr1_35636303‐35641236 +	366	40.48	5.27	−0.378	Cytoplasmic
Erasers	LOC_Os01g66100.1	OsALKBH‐11	Chr1_38382382‐38385504 +	389	42.51	5.73	−0.248	Plasma membrane
Erasers	LOC_Os01g70930.1	OsALKBH‐12	Chr1_41057016‐41058872 −	366	40.74	5.01	−0.315	Cytoplasmic
Erasers	LOC_Os02g17940.1	OsALKBH‐13	Chr2_10386299‐10390290 +	351	40.12	5.1	−0.425	Cytoplasmic
Erasers	LOC_Os02g37890.1	OsALKBH‐14	Chr2_22900713‐22906465 −	442	49.27	5.99	0.174	Plasma membrane
Erasers	LOC_Os02g41954.1	OsALKBH‐15	Chr2_25199386‐25203742 −	359	39.02	5.57	−0.17	Extracellular
Erasers	LOC_Os02g52840.1	OsALKBH‐16	Chr2_32310799‐32312184 −	331	36.82	6.01	−0.335	Cytoplasmic
Erasers	LOC_Os02g53180.1	OsALKBH‐17	Chr2_32558635‐32562705 +	344	38.29	6.81	−0.368	Mitochondrial
Erasers	LOC_Os02g58070.1	OsALKBH‐18	Chr2_35545505‐35548352 +	310	34.27	8.85	−0.324	Chloroplast
Erasers	LOC_Os03g03034.1	OsALKBH‐19	Chr3_1235373‐1245060 −	342	38.74	6.42	−0.344	Cytoplasmic
Erasers	LOC_Os03g06990.1	OsALKBH‐20	Chr3_3566777‐3568821 +	277	30.23	5.17	−0.148	Extracellular
Erasers	LOC_Os03g07000.1	OsALKBH‐21	Chr3_3569977‐3571884 +	299	32.76	6.61	−0.325	Extracellular
Erasers	LOC_Os03g18030.1	OsALKBH‐22	Chr3_10038840‐10044429 −	387	41.50	6.47	−0.214	Chloroplast
Erasers	LOC_Os03g32470.1	OsALKBH‐23	Chr3_18570651‐18572190 +	372	41.60	5	−0.342	Cytoplasmic
Erasers	LOC_Os03g42130.1	OsALKBH‐24	Chr3_23450164‐23452171 −	352	39.29	5.17	−0.405	Cytoplasmic
Erasers	LOC_Os03g48430.1	OsALKBH‐25	Chr3_27580909‐27582212 −	373	39.58	5.8	−0.137	Cytoplasmic
Erasers	LOC_Os03g55380.1	OsALKBH‐26	Chr3_31503703‐31507753 +	310	33.88	6.47	−0.354	Nuclear
Erasers	LOC_Os03g58890.1	OsALKBH‐27	Chr3_33522519‐33524525 −	297	32.98	6.59	−0.313	Extracellular
Erasers	LOC_Os03g63900.1	OsALKBH‐28	Chr3_36103513‐36105068 +	362	40.79	5.97	−0.318	Mitochondrial
Erasers	LOC_Os03g63970.1	OsALKBH‐29	Chr3_36150664‐36152517 +	372	42.26	5.98	−0.358	Mitochondrial
Erasers	LOC_Os03g64280.1	OsALKBH‐30	Chr3_36318345‐36319662 +	369	40.25	6.05	−0.183	Cytoplasmic
Erasers	LOC_Os04g10350.1	OsALKBH‐31	Chr4_5590325‐5592699 +	375	40.78	5.54	−0.178	Mitochondrial
Erasers	LOC_Os04g27850.1	OsALKBH‐32	Chr4_16449496‐16456516 +	267	29.99	7.68	−0.246	Extracellular
Erasers	LOC_Os04g33360.1	OsALKBH‐33	Chr4_20200072‐20201885 +	371	40.23	6.27	−0.286	Chloroplast
Erasers	LOC_Os04g44150.1	OsALKBH‐34	Chr4_26130707‐26134356 −	358	39.04	7.04	−0.209	Mitochondrial
Erasers	LOC_Os04g49194.1	OsALKBH‐35	Chr4_29331563‐29338265 +	340	38.82	5.46	−0.394	Cytoplasmic
Erasers	LOC_Os04g49210.1	OsALKBH‐36	Chr4_29338950‐29341166 −	352	39.19	5.94	−0.297	Nuclear
Erasers	LOC_Os04g55070.1	OsALKBH‐37	Chr4_32747192‐32750831 −	326	35.80	5.34	−0.247	Cytoplasmic
Erasers	LOC_Os04g56700.1	OsALKBH‐38	Chr4_33809347‐33812300 −	377	41.56	5.66	−0.251	Cytoplasmic
Erasers	LOC_Os04g57160.1	OsALKBH‐39	Chr4_34060040‐34063474 +	326	36.84	5.87	−0.358	Cytoplasmic
Erasers	LOC_Os04g57180.1	OsALKBH‐40	Chr4_34067653‐34070800 −	333	37.19	5.96	−0.346	Nuclear
Erasers	LOC_Os05g03640.1	OsALKBH‐41	Chr5_1561980‐1568165 −	368	40.30	5.95	−0.299	Mitochondrial
Erasers	LOC_Os05g05680.1	OsALKBH‐42	Chr5_2825323‐2826992 +	308	34.58	5.11	−0.452	Cytoplasmic
Erasers	LOC_Os05g06670.1	OsALKBH‐43	Chr5_3459616‐3465991 +	382	40.62	6.63	−0.051	Chloroplast
Erasers	LOC_Os05g08540.1	OsALKBH‐44	Chr5_4660662‐4662589 −	384	41.56	5.96	−0.195	Chloroplast
Erasers	LOC_Os05g11810.1	OsALKBH‐45	Chr5_6720998‐6726973 −	378	39.33	9.37	−0.46	Nuclear
Erasers	LOC_Os05g34854.1	OsALKBH‐46	Chr5_20685346‐20692274 −	445	47.63	6.7	−0.13	Chloroplast
Erasers	LOC_Os05g41010.1	OsALKBH‐47	Chr5_24040970‐24043906 +	319	34.29	6.51	−0.287	Extracellular
Erasers	LOC_Os05g43880.1	OsALKBH‐48	Chr5_25515053‐25516204 −	354	37.79	6.37	−0.104	Chloroplast
Erasers	LOC_Os05g48230.1	OsALKBH‐49	Chr5_27643114‐27647843 +	404	45.26	8.57	−0.493	Mitochondrial
Erasers	LOC_Os05g48700.1	OsALKBH‐50	Chr5_27910731‐27912924 −	353	37.31	6.02	−0.091	Chloroplast
Erasers	LOC_Os05g50090.1	OsALKBH‐51	Chr5_28705905‐28708523 −	384	41.99	5.72	−0.308	Cytoplasmic
Erasers	LOC_Os06g06720.1	OsALKBH‐52	Chr6_3145466‐3147618 +	352	38.63	5.65	−0.271	Mitochondrial
Erasers	LOC_Os06g07914.1	OsALKBH‐53	Chr6_3846106‐3848325 +	307	34.03	4.84	−0.306	Cytoplasmic
Erasers	LOC_Os06g07932.1	OsALKBH‐54	Chr6_3854646‐3857393 +	350	39.93	5.16	−0.399	Cytoplasmic
Erasers	LOC_Os06g07941.1	OsALKBH‐55	Chr6_3858434‐3860468 +	317	35.58	4.97	−0.234	Cytoplasmic
Erasers	LOC_Os06g08014.1	OsALKBH‐56	Chr6_3884192‐3885867 +	352	39.10	4.75	−0.28	Cytoplasmic
Erasers	LOC_Os06g08023.1	OsALKBH‐57	Chr6_3889190‐3892359 +	375	42.83	5.28	−0.271	Cytoplasmic
Erasers	LOC_Os06g08032.1	OsALKBH‐58	Chr6_3893672‐3895400 +	350	39.93	5.16	−0.399	Cytoplasmic
Erasers	LOC_Os06g08041.1	OsALKBH‐59	Chr6_3896490‐3898785 +	317	35.58	4.97	−0.234	Cytoplasmic
Erasers	LOC_Os06g08060.1	OsALKBH‐60	Chr6_3900488‐3903056 +	354	39.71	5.41	−0.269	Cytoplasmic
Erasers	LOC_Os06g14390.1	OsALKBH‐61	Chr6_8031719‐8035243 −	365	39.17	5.23	−0.018	Chloroplast
Erasers	LOC_Os06g14400.1	OsALKBH‐62	Chr6_8038363‐8041831 −	365	38.91	6.01	0.044	Chloroplast
Erasers	LOC_Os06g37590.1	OsALKBH‐63	Chr6_22258061‐22259545 −	293	33.62	5.88	−0.423	Cytoplasmic
Erasers	LOC_Os06g42130.1	OsALKBH‐64	Chr6_25295991‐25297316 −	373	40.71	5.68	−0.207	Cytoplasmic
Erasers	LOC_Os07g01340.1	OsALKBH‐65	Chr7_213778‐219544 +	341	38.69	5.87	−0.31	Cytoplasmic
Erasers	LOC_Os07g07410.1	OsALKBH‐66	Chr7_3699591‐3701560 +	369	41.12	6.32	−0.202	Mitochondrial
Erasers	LOC_Os07g07420.1	OsALKBH‐67	Chr7_3702287‐3705030 +	367	40.49	5.75	−0.188	Extracellular
Erasers	LOC_Os07g09630.1	OsALKBH‐68	Chr7_5116627‐5119153 −	319	35.18	8.02	−0.266	Extracellular
Erasers	LOC_Os08g15149.1	OsALKBH‐69	Chr8_9165968‐9167484 −	296	32.98	5.3	−0.299	Cytoplasmic
Erasers	LOC_Os08g30080.1	OsALKBH‐70	Chr8_18491723‐18493264 −	396	42.49	5.55	−0.092	Cytoplasmic
Erasers	LOC_Os08g30100.1	OsALKBH‐71	Chr8_18509005‐18510244 −	374	39.90	5.45	−0.031	Cytoplasmic
Erasers	LOC_Os08g30150.1	OsALKBH‐72	Chr8_18542133‐18543586 −	328	35.00	6.51	−0.121	Chloroplast
Erasers	LOC_Os08g30210.1	OsALKBH‐73	Chr8_18568971‐18570352 −	376	40.20	5.45	−0.077	Cytoplasmic
Erasers	LOC_Os08g32160.1	OsALKBH‐74	Chr8_19950552‐19952391 −	311	33.73	5.3	−0.214	Cytoplasmic
Erasers	LOC_Os08g32170.1	OsALKBH‐75	Chr8_19954661‐19956231 −	314	34.19	5.34	−0.232	Cytoplasmic
Erasers	LOC_Os08g37456.1	OsALKBH‐76	Chr8_23723612‐23727247 −	337	37.00	5.53	−0.161	Cytoplasmic
Erasers	LOC_Os08g44590.1	OsALKBH‐77	Chr8_28043209‐28045588 +	383	41.83	5.96	−0.135	Chloroplast
Erasers	LOC_Os09g07020.1	OsALKBH‐78	Chr9_3404354‐3408176 −	435	48.94	7.1	−0.315	Chloroplast
Erasers	LOC_Os09g18450.1	OsALKBH‐79	Chr9_11309063‐11310776 +	331	37.08	6.87	−0.265	Cytoplasmic
Erasers	LOC_Os09g18470.1	OsALKBH‐80	Chr9_11320295‐11322989 +	282	31.47	5.7	−0.135	Cytoplasmic
Erasers	LOC_Os09g18520.1	OsALKBH‐81	Chr9_11348302‐11349993 −	236	26.60	6.39	−0.348	Cytoplasmic
Erasers	LOC_Os09g27750.1	OsALKBH‐82	Chr9_16886019‐16887617 −	322	36.34	5.2	−0.315	Cytoplasmic
Erasers	LOC_Os09g27820.1	OsALKBH‐83	Chr9_16925457‐16927082 +	322	36.44	4.99	−0.352	Cytoplasmic
Erasers	LOC_Os09g39720.1	OsALKBH‐84	Chr9_22790611‐22792019 +	378	41.62	6.27	−0.084	Mitochondrial
Erasers	LOC_Os10g14180.1	OsALKBH‐85	Chr10_7692650‐7696843 +	465	51.03	6.44	−0.29	Mitochondrial
Erasers	LOC_Os10g35470.1	OsALKBH‐86	Chr10_18981326‐18985212 +	321	34.26	9.37	−0.313	Chloroplast
Erasers	LOC_Os10g37880.1	OsALKBH‐87	Chr10_20281431‐20285018 +	308	34.24	5.35	−0.25	Cytoplasmic
Erasers	LOC_Os10g37899.1	OsALKBH‐88	Chr10_20293206‐20299471 +	544	59.36	6.66	−0.363	Chloroplast
Erasers	LOC_Os10g39140.1	OsALKBH‐89	Chr10_20886352‐20892217 +	342	38.73	5.59	−0.378	Cytoplasmic
Erasers	LOC_Os10g40880.1	OsALKBH‐90	Chr10_21968925‐21970501 −	350	39.67	6.23	−0.387	Cytoplasmic
Erasers	LOC_Os10g40900.1	OsALKBH‐91	Chr10_21979109‐21981920 −	428	47.39	5.65	−0.612	Nuclear
Erasers	LOC_Os10g40934.1	OsALKBH‐92	Chr10_21988523‐21993289 −	362	41.04	5.58	−0.295	Cytoplasmic
Erasers	LOC_Os10g41020.1	OsALKBH‐93	Chr10_22019715‐22023394 −	360	40.37	5.07	−0.221	Cytoplasmic
Erasers	LOC_Os11g08380.1	OsALKBH‐94	Chr11_4415910‐4418435 −	309	34.75	4.93	−0.403	Cytoplasmic
Erasers	LOC_Os11g25060.1	OsALKBH‐95	Chr11_14285137‐14290853 +	391	42.80	6.12	−0.27	Extracellular

### 3.2 Conserved Motifs and Gene Structure Analysis of Osm6As

**Figure 1:**
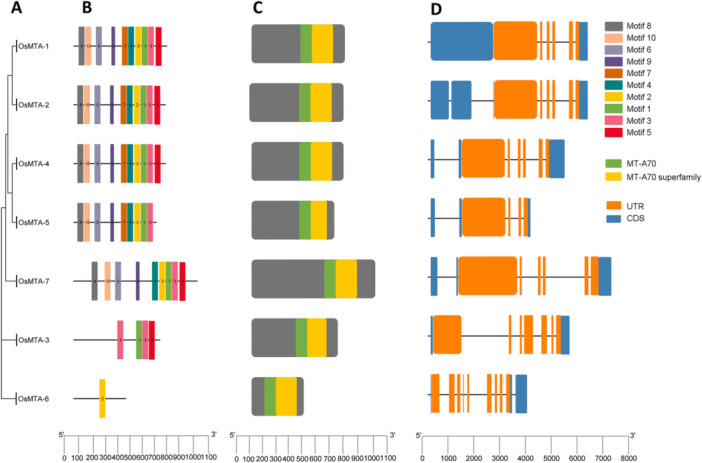
Illustrates the motif distributions and gene structures of the Osm6A writers (MT‐A70 superfamily). (A) The phylogenetic tree represents the relationships among m6A writers. (B) The motif composition of the m6A writer in the rice is depicted with motifs 1–10 highlighted in various colors. (C) The visualization of conserved domains within m6A writer proteins. (D) The exon/intron organization of m6A writer genes is shown, where blue boxes represent exons and the black lines indicate introns. Orange boxes represent the untranslated regions (UTRs). The sizes of exons and introns can be assessed using the scale at the bottom, and the relative positions are depicted proportionally on a kilobase scale at the bottom of the figure.

The discovery motifs also served various biological functions, and their detection and characterization are crucial for investigating molecular mechanisms within cells, including gene expression and its regulation [49]. The structure and functional analysis of protein sequences associated with Osm6A modifiers revealed both conserved and diverse features. The phylogenetic analysis classified these genes into distinct clades, showing evolutionary divergence (Figure 1A). Conserved motif analysis identified that most Osm6A writers contained 8–10 motifs, particularly motifs 1, 2, 3, and 5, which are associated with methyltransferase activity (Figure 1B). All members were detected to contain core MT‐A70 and MT‐A70 superfamily domains, consistent with their involvement as m6A methyltransferases (Figure 1C). Proteins are fundamental to various biochemical reactions within organisms and are encoded by exons. Consequently, introns play an essential role in gene expression and its regulation. The distribution of exons and introns is a significant characteristic in the functional characterization of a gene [50]. Gene structure analysis revealed that the exons ranged from 2 to 11. Although OsMTA‐1, OsMTA‐2, OsMTA‐4, and OsMTA‐5 each contained 10–11 exons and 9–10 introns. These results suggest more complex transcriptional regulations. In addition, OsMTA‐3 had 4 exons and 3 introns, and OsMTA‐7 exhibited an intermediate structure with 7 exons and 6 introns. Subsequently, the OsMTA‐6 had the simplest structure, consisting of 2 exons and 1 intron (Figure 1D). These results suggest that the diversity in exon–intron organization may relate to differential gene expression and function within the Osm6A writer gene family.

**Figure 2:**
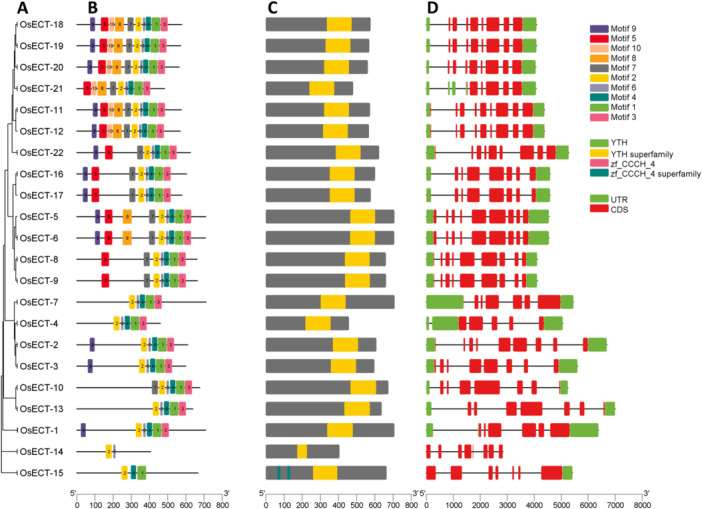
Illustrates the motif distributions and gene structures of the Osm6A readers (YTH superfamily). (A) The phylogenetic tree represents the relationships among Osm6A readers. (B) The motif composition of m6A readers in the rice *Oryza sativa* is depicted with motifs 1–10 highlighted in various colors. (C) The visualization of conserved domains within m6A reader proteins. (D) The exon/intron organization of m6A reader genes is shown, where red boxes represent exons and the black lines indicate introns. Green boxes represent the untranslated regions (UTRs). The sizes of exons and introns can be assessed using the scale at the bottom, and the relative positions are depicted proportionally on a kilobase scale at the bottom of the figure.

In addition, the structural and functional analysis of protein sequences associated with m6A reader elucidates their complex role in *Oryza sativa*. The phylogenetic relationship among OsECT members underscores distinct evolutionary divergence, implying potential functions within the YTH family (Figure 2A). The 10 conserved motifs across the m6A reader OsECT member repertoire reveal a significant range in motif compositions, with individual proteins harboring a minimum of 2–3 motifs, for example, OsECT‐8, OsECT‐15, to a maximum of 8–10 motifs, similarly OsECT‐18, OsECT‐19, OsECT‐20, highlighting structural variations that may underline functional specificity (Figure 2B). The domain architecture of these proteins consistently demonstrates the presence of YTH and YTH superfamily domains, thereby confirming their crucial role in m6A modifier recognition. However, specific co‐occurring zf_CCCH_4 and zf_CCCH_4 superfamily domains are observed within the same OsECTs proteins. This synergistic domain organization proposes a novel regulatory paradigm in which m6A modifier recognition is intricately coupled with supplementary RNA‐binding specificities or PPI interfaces. Such a pleiotropic function implies heightened control over RNA fate, potentially modulating stability, subcellular localization, and translational efficiency (Figure 2C). Furthermore, the gene structure analysis reveals substantial variability in exonic range from a solitary exon to approximately 10, and intronic range, underscoring the evolutionary pliability and the inherent capacity to generate functionally divergent protein isoforms via alternative splicing mechanisms (Figure 2D). Collectively, these findings provide a robust milieu for understanding the intricate epitranscriptomic insight in rice, implicating a higher‐order regulatory complexity mediated by the OsECT proteins.

Beyond the comprehensive analysis of m6A “writer”, “readers” proteins, we also investigate the complete understanding of m6A reversibility by the “erasers” enzyme. These demethylases are crucial for dynamically modulating m6A modifiers, thereby influencing RNA fate and cellular processes. The most prominent protein domains identified within the OsALKBH family are the 2OG‐Fell_Oxy (2‐oxoglutarate and iron (II)‐dependent oxygenase) and DIOX_N (Dioxygenase N‐terminal) domains. These domains are characteristic of the (ALKBH) Alkylated DNA repair protein homolog superfamily, which functions as non‐heme Fe(II)‐dependent dioxygenases known to catalyze oxidative demethylation reactions on various nucleic acid substrates, including m6A on RNA [51, 52]. In this study, the detection of ubiquity in the OsALKBH gene set firmly implicates these proteins as candidates for m6A demethylases, also known as “erasers”, in rice *(Oryza sativa*). Furthermore, the co‐occurrence of diverse domains such as the Pkinase superfamily (protein kinase superfamily), HGWLP, and SHK domains within certain OsALKBH members suggested sophisticated functional integration (Supporting Information: Figure S1A–D). While the core enzymatic activity resides in the oxygenase domains, the additional modules in our study could confer novel regulatory layers that mediate phosphorylation‐dependent modulation of demethylase activity, scaffolding protein complexes, or even exhibiting moonlighting functions. Collectively, our findings significantly advance our understanding of the complete m6A epitranscriptomic mechanism in rice. The identified OsMTA, “writers”, OsECT “readers”, and OsALKBH “erasers” represent critical components governing the dynamic and reversible nature of m6A modification, and elucidating the precise interplay between writers, readers, and erasers is paramount for unraveling the intricate regulatory layers that influence gene expression, development, and abiotic stress responses in rice *Oryza sativa*.

### 3.3 Phylogenetics and Comparative Gene Duplication Analysis

**Figure 3:**
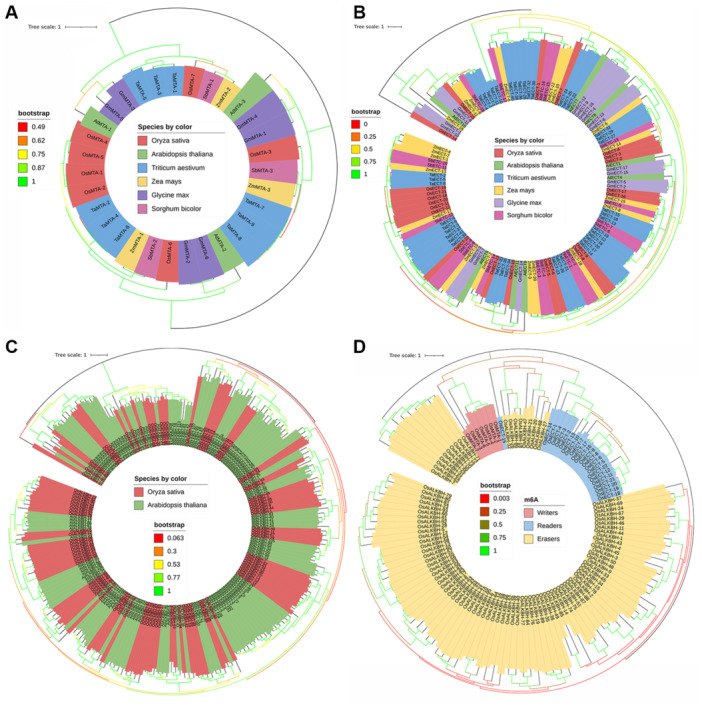
The phylogenetic relationship among m6A‐related genes in dicot and monocot species, categorized into three groups: (A) Writers, (B) Readers, (C) Erasers, and (D) compiles all 124 m6A protein families. The phylogenetic tree was constructed using the Maximum Likelihood Method in MEGA 12, incorporating a bootstrap value of 1000.

Determining the evolutionary relationships of gene families across species is crucial for detecting gene ancestries, examining their evolution and molecular adaptation, and understanding morphological traits [53]. A phylogenetic analysis was conducted using protein sequences of m6A modifiers from dicot and monocot species. Consequently, we retrieve m6A‐related “writer” genes from *Arabidopsis thaliana*, 3 genes from *Triticum aestivum*, 9 genes from *Zea mays*, 3 genes from *Sorghum bicolor*, 3 genes from *Glycine max*, 6 genes, including *Oryza sativa*, to create a dataset as described by [54]. Notably, an orthologous relationship was observed among specific m6A‐related genes from (*Triticum aestivum*, *Zea mays*, *Sorghum bicolor*), which exhibit mixed clades, for example, TaMTA‐2, ZmMTA‐2, and SbMTA‐2 (Figure 3A). These results suggest evolutionary conservation of methyltransferase functions. Furthermore, dicot species clades, such as GmMTA and AtMTA genes, show distinct evolutionary patterns.

A total of 126 m6A readers genes were used to construct phylogenetic relationships among them *Arabidopsis thaliana*, 8 genes such as *ECT5, ECT1, ECT8, ECT4, ECT7, ECT10, ECT9, ECT11*, from *Triticum aestivum*, 41 genes, *Zea mays*, 20 genes, *Sorghum bicolor* 18 genes, *Glycine max* 17 genes, and *Oryza sativa* 22 genes (Figure 3B). Notably, the majority of m6A‐related genes from monocot species, such as TaECT, OsECT, ZmECT, and SbECT, cluster together, forming large, often intermingled clades. Subsequently, the AtETC genes typically exhibit smaller, more distinct clades, underscoring their evolutionary divergence from monocot species. This divergence indicates species‐specific functional mechanisms within the m6A eraser family. To elucidate the evolutionary relationships of m6As “erasers” within the ALKBH family, 113 genes from *Arabidopsis thaliana*, along with our identified 95 genes from *Oryza sativa*, were used to construct a phylogenetic analysis (Figure 3C). Subsequently, all 124 identified m6A across their subgroups, such as writers, readers, and erasers, were utilized to construct phylogenetic relationships (Figure 3D). Our analysis showed that all m6A‐related genes were classified into three distinct groups based on their roles in m6A modification. The writer and the reader genes belong to separate clades, with high bootstrap values indicating significant evolutionary divergence and stability within each group. Whereas the eraser group represents the large family, suggesting potentially high levels of functional diversification of m6A‐modifiers in the *Oryza sativa* lineage. These results indicate that erasers, a demethylation group of m6A modifiers, can be distinctly classified into three primary functions. Specifically, eraser genes that share clades with writers and readers might show similar functional roles.

### 3.4 Cis‐Acting Elements Analysis of Osm6A Writers, Readers, and Erasers

**Figure 4:**
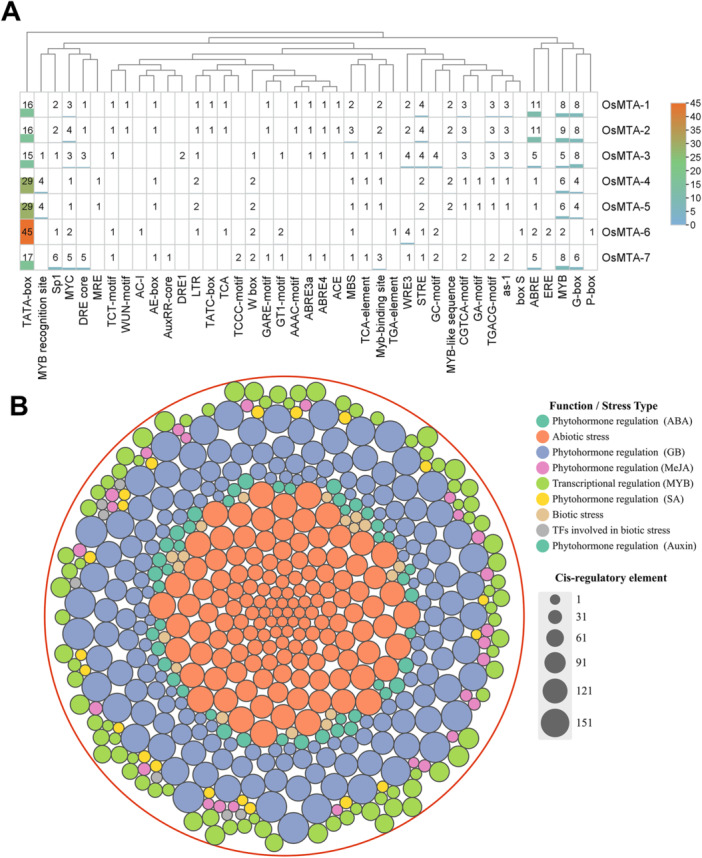
The analysis of cis‐regulatory elements within the 2000 bp region upstream of the transcription start site of Osm6A writer genes was conducted, accompanied by an illustration depicting their distribution along the promoter sequence. (A) The number of cis elements identified for each functional category was recorded, along with the corresponding data obtained from the PlantCARE database. The x‐axis denoted each gene, and the corresponding numeric values indicated the number of identified motifs. The y‐axis lists the distinct motif names identified within the Osm6A writer subgroup. The color scale denotes abundance, with orange indicating a high number, from sky blue to green representing a low or absent motif in the heatmap. (B) The cis‐regulatory elements were categorized by functional significance, including associations with hormonal signals, abiotic stress, biotic stress, and responsiveness to transcription factors (TFs), with each category represented by distinct colors.

Gene expression is precisely regulated during stress responses and developmental processes, which are controlled by cis‐regulatory elements such as promoters and enhancers [55]. In this study, we retrieved cis‐regulatory elements were classified into several categories based on their function and positive regulatory roles, including phytohormone‐responsive elements such as those for ABA, auxin, gibberellin, MeJA, SA, and elements related to abiotic stress, biotic stress, MYB‐related TFs, and TFs involved in biotic interactions (Figure 4A,B) and (Supporting Information: Figure S2). Notably, the abundance of TATA‐box motifs was found in all Osm6A writers, whereas OsMTA‐6 detected the most cis‐element motifs, suggesting that these genes are functionally involved with TATA‐box‐binding proteins (TBPs), which exhibit distinct patterns of gene expression. Our results indicate that the elements are present in all Osm6A writer genes (Supporting Information: Table S4). These findings suggest that all these elements play a role in regulating plant growth and development, particularly in response to abiotic stress.

**Figure 5:**
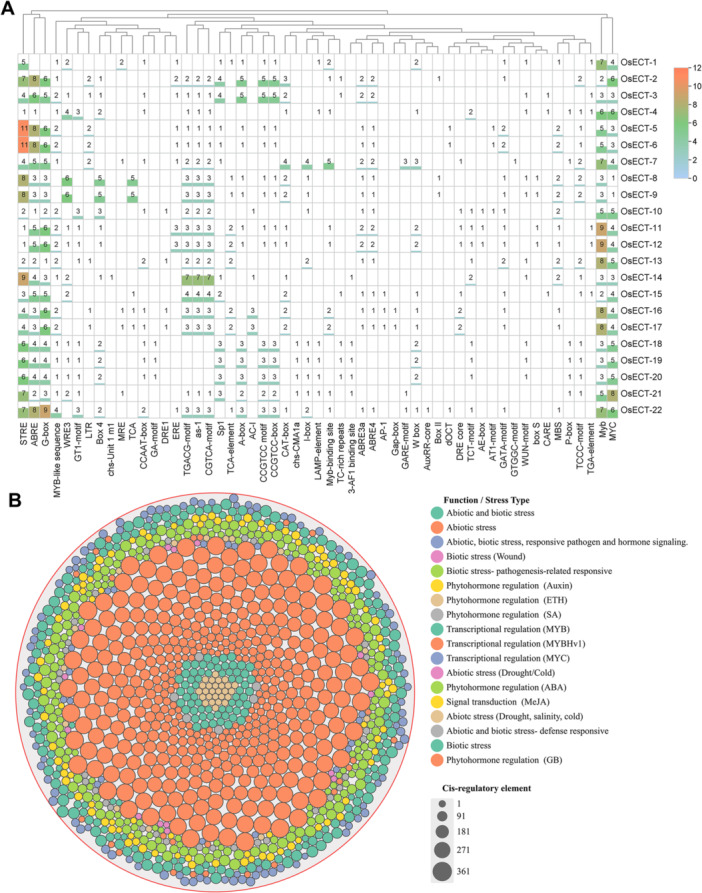
The analysis of cis‐regulatory elements within the 2000 bp upstream region of the transcription start site of the Osm6A reader genes. (A) A quantitative heatmap shows the number of cis‐regulatory elements identified within each gene and their functional group, with annotations retrieved from the PlantCARE database. The x‐axis denoted each gene, and the corresponding numeric values indicated the number of identified motifs. The y‐axis lists the distinct motif names identified within the Osm6A reader subgroup. The color scale denotes abundance, with orange indicating a high number, from sky blue to green representing low or absent motif in the heatmap. (B) A distribution illustrated each gene and its identified cis‐regulatory elements across the promoter regions, organized by their functional relevance, which includes hormonal responsiveness, abiotic stress, biotic stress, and transcription factors (TFs), binding sites. Each category is color‐coded for visual distinction.

Promoter analyses of YTH family m6A readers revealed a diverse array of cis elements associated with responses to abiotic stress, phytohormone signaling, and the regulation of plant growth and development. Notably, OsECT‐2 exhibited a significantly enriched promoter architecture, reinforcing its role as a multi‐stress‐responsive m6A reader protein. Furthermore, the key abiotic stress‐responsive elements identified in OsECT‐2 include ABRE, ABRE3a, and ABRE4, which facilitate ABA‐dependent transcriptional activation under salt and drought conditions, as well as LTR, which is linked to cold responsiveness. Additionally, STRE and TC‐rich repeats suggest that OsECT‐2 is involved in general stress responses. The presence of MYB, MYB‐like, and MYC‐binding sites indicates regulation by stress‐inducible TFs, which are crucial for response to salt and dehydration stress. The highest motifs identified were associated with abiotic stress and ABA‐responsive elements (Figure 5A,B) and (Supporting Information: Figure S3). Importantly, all genes identified from the YTH‐domain superfamily in this study contained the cis‐acting regulatory elements, underscoring their involvement in multi‐stress regulatory functions (Supporting Information: Table S5).

**Figure 6:**
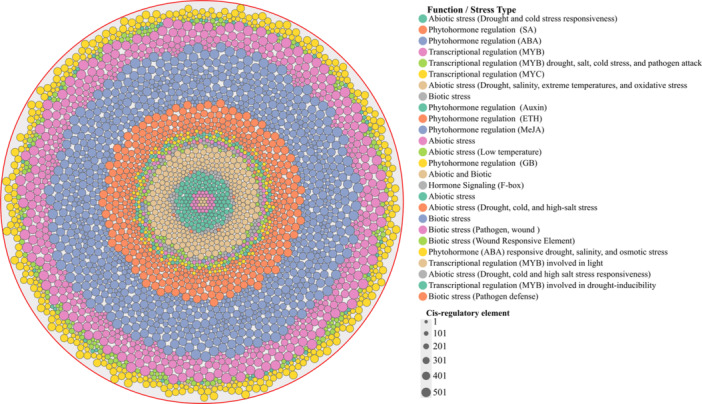
The distribution analysis of cis‐elements within each functional stress group. Illustrated the number of motifs identified in the Osm6A eraser genes. The motifs were determined using data from the PlantCARE database.

All 95 identified genes from the m6A eraser underwent promoter analysis, revealing the presence of specific stress and hormone‐responsive elements. These included elements responsive to abiotic stress such as drought, salinity, osmotic stress, extreme temperatures (low and high), and oxidative stress. Additionally, we identified phytohormone regulatory elements responsive to ABA for drought, salinity, and osmotic stress, as well as SA, Auxin, ethylene (ETH), methyl jasmonate (MeJA), gibberellin (GB), and hormone signaling elements associated with F‐box proteins. TFs elements, particularly those involved in transcriptional regulation (MYB and MYC), were also noted, highlighting their roles in light responsiveness, drought‐inducibility, and biotic responses, including pathogen attack and wound responses, as illustrated in (Figure 6) and (Supporting Information: Figure S4). Notably, the analysis of promoters from m6A erasers revealed that phytohormone regulatory elements were the most abundant, followed by abiotic stress cis‐regulatory elements in second place (Supporting Information: Table S6). These findings suggest that abiotic, biotic, and hormonal factors, including TFs, play a significant role in regulating all identified m6A genes, writer, reader, and eraser, thereby influencing growth and development in rice (*Oryza sativa*), particularly under abiotic stress conditions. The identified stress‐associated elements, such as drought, salinity, extreme temperatures, oxidative stress, wound responses, and pathogen attacks, along with MYC, MYB TFs, and F‐box hormonal signals, underscore the intricate regulatory network involved in stress‐induced mechanisms (Supporting Information: Figure S5). It is noteworthy that the elements identified in each Osm6A gene indicate their potential influence and multifunctionality in regulating growth, development, and stress responses in rice. Collectively, these findings demonstrate a significant relationship between promoter architecture and functional significance, suggesting that Osm6A genes, particularly OsECT‐2, are transcriptionally poised to respond to abiotic stress through cis‐acting elements that drive activation. Our results support its role as a critical epitranscriptomic regulator in enhancing rice salt tolerance.

### 3.5 Gene Duplicate Events, Chromosomal Location, and Collinear Analysis of osm6As

**Figure 7:**
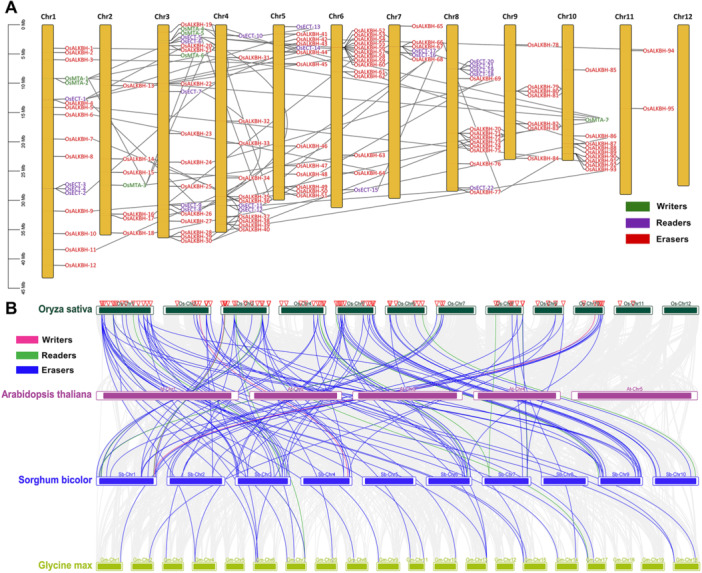
The genomic localization and distribution patterns of the Osm6As genes within the rice (*Oryza sativa*) genome were analyzed. (A) The left scale indicates the chromosome lengths in (Mb). A total of 124 Osm6A genes are distributed across 11 chromosomes. Each subgroup of Osm6A identified on the chromosome is represented by distinct color fonts: writers (green), readers (purple), and erasers (red), respectively. The collinear blocks were depicted by gray lines in the background. (B) The genome‐scale analysis of multi‐synteny and collinear relationships among m6A‐related genes across dicot and monocot species, specifically *Oryza sativa, Arabidopsis thaliana, Sorghum bicolor*, and *Glycine max*, reveals important insight. The gray background lines represent the collinear blocks between dicot and monocot species, whereas the colored lines indicate the segmental duplications of the m6A‐related genes: writers (green), readers (purple), and erasers (red), respectively. The red triangles denote the location Osm6As within the genome.

Gene duplication is considered a significant force in expanding gene families, including tandem duplication, fragment duplication, and whole‐genome duplication [56]. To elucidate the chromosome location and distribution of Osm6As, the identified gene from all subgroups, likely writers, readers, and erasers, were mapped to the rice *Oryza sativa* genome. A total of 124 Osm6As genes were identified and mapped across 11 chromosomes, no genes were found on chromosome 12. The gene duplication analysis revealed Chr 1 and Chr 3 had the most abundant Osm6As genes. Specifically, OsMTA‐1, OsMTA‐2, were mapped in Chr 1, OsMTA‐3 was mapped on Chr 2, OsMTA‐4, OsMTA‐5, and OsMTA‐6 were mapped on Chr 4, while OsMTA‐7 was mapped on Chr 10. Subsequently, Osm6As reader (OsECTs) were mapped on chromosomes 1, 3, 4, 5, 7, and 8. Moreover, Osm6As erasers (OsALKBHs) were mapped on 11 chromosomes (Figure 7A). Notably, the disposition of Osm6As genes suggests the presence of genomic regions that may have undergone tandem duplication, leading to significantly uneven diversification and functional expansion within these gene families.

To investigate the expansion of gene families associated with m6A, an in‐depth comparative genomic analysis was conducted through multi‐synteny analysis between monocots and dicots. The MCScanX tool, recognized for its efficiency in detecting and analyzing gene synteny and collinearity [57], was utilized to elucidate the collinear relationships of m6A‐related genes. This study performed a comparative synteny and collinearity analysis among *Oryza sativa* and three model species: *Arabidopsis thaliana*, *Sorghum bicolor*, and *Glycine max*, thereby enhancing the understanding of gene families linked to the mRNA modification pathway (Figure 7B). The results indicated a significant number of syntenic gene pairs between rice and sorghum, supporting the close evolutionary relationship characteristic of monocots. This finding suggests that the conserved regions likely originated from segmental duplication (SD) or whole genome duplication (WGD) events in the common ancestor of monocots. In contrast, a reduced number of collinear gene pairs were identified between rice and Arabidopsis, a distantly related dicot, reflecting amplified divergence and potential gene loss or neofunctionalization over evolutionary time. Notably, in this study, rice shared a considerable number of syntenic pairs with *Glycine max*, indicating multiple rounds of WGD in soybean. A total of 124 orthologous pairs were observed, with only two duplicated gene pairs identified between rice and Arabidopsis. Despite this limited number, these pairs highlight the evolutionary conservation of certain m6A regulators across monocot and dicot lineages, suggesting that these conserved orthologs may represent core components of the m6A machinery preserved throughout extensive evolutionary divergence. Moreover, 34 gene pairs were identified between rice and *Glycine max*, likely reflecting the polyploid nature and WGD events in soybeans, which have amplified many gene families involved in RNA modifications. Notably, the most extensive duplication pattern was observed between *Oryza sativa* and *Sorghum bicolor*, with a total of 89 syntenic gene pairs. This strong collinearity is attributed to the close evolutionary relationship between these two monocot species, which share a more recent common ancestor than dicots. Collectively, these results suggest that m6A‐related genes exhibit differential expression across species, with conservation in Arabidopsis, polyploidy‐driven amplification in soybean, and strong conservation in sorghum, underscoring their fundamental biological roles in plant development and stress adaptation.

### 3.6 RNA‐Seq and Gene Expression Analysis of Osm6As

**Figure 8:**
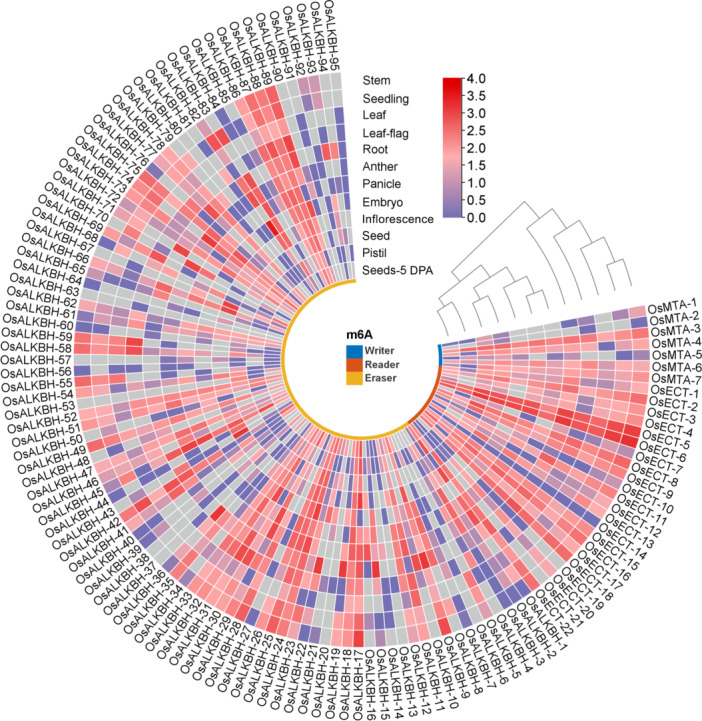
The circular heatmap illustrates the tissue‐specific expression of m6A regulatory genes in rice. The inner ring color indicates the three subgroups of m6A regulators: writers (blue), readers (red), and erasers (yellow), based on RNA‐seq data from various rice tissues. The heatmap column represents gene expression levels, while the rows display distinct tissue clusters, with normalized data. The color gradients indicate relative expression levels, with red denoting high expression and purple/gray indicating low or absent expression.

To predict gene functions, which are essential for understanding the underlying molecular mechanisms of gene expression, RNA‐seq serves as a powerful tool that plays a crucial role in uncovering integrated gene expression [58]. To investigate the critical role of m6A‐related genes in rice, we utilized RNA‐seq from various tissues using TPM. The analysis revealed differential expressions of m6A‐related genes (writer, reader, and eraser), with the writer gene OsMTA‐3 exhibiting significant expression across various tissues, including stem, seedling, leaf, leaf‐flag, root, anther, panicle, embryo, inflorescence, seed, pistil, and seeds at 5 DPA. However, expression levels of OsMTA‐1 and OsMTA‐2 were notably lower; for instance, OsMTA‐2 showed no expression in the seedling, leaf, root, anther, embryo, pistil, and seed at 5 DPA. Conversely, OsMTA‐3, OsMTA‐4, OsMTA‐6, and OsMTA‐7 were expressed at high levels across all tissues. In contrast, OsMTA‐1, OsMTA‐2, and OsMTA‐5 displayed lower expression, with tissue showing no expression, as shown in Figure 8. Notably, several m6A write genes, including OsMTA‐1, OsMTA‐3, and OsMTA‐5, exhibited relatively high expression in seedlings, leaf, and stems. These results suggest that these writer genes may play a critical role during the early stages of vegetative growth, particularly in cell division and elongation. Additionally, among the reader genes, broad tissue expressions were observed across all samples in OsECT‐5 and OsECT‐6. This pattern suggests a potential specialized role for these genes in decoding m6A pathways during both vegetative and reproductive stages. However, several reader genes exhibited limited or no expression (indicated by gray color) in specific tissues, such as pistils or seeds at 5 DPA, suggesting tissue‐specific regulation. Furthermore, the eraser group (OsALKBH family) displayed more diverse tissue expression profiles. Notably, genes such as OsALKBH‐1, OsALKBH‐7, and OsALKBH‐19 were highly expressed in roots, leaf, and seedlings, indicating their involvement in m6A demethylation during active growth or in response to environmental stimuli. Conversely, several genes, including OsALKBH‐37, OsALKBH‐60, OsALKBH‐76, OsALKBH‐80, OsALKBH‐90, and OsALKBH‐95, exhibited low or no expression in most tissues, possibly indicating specialized or dormant roles. In contrast, the overall tissue‐expression analysis provides strong evidence that writer and reader genes are highly expressed and may play essential roles throughout development, whereas eraser genes exhibit a more selective role, potentially reflecting functional specialization.

**Figure 9:**
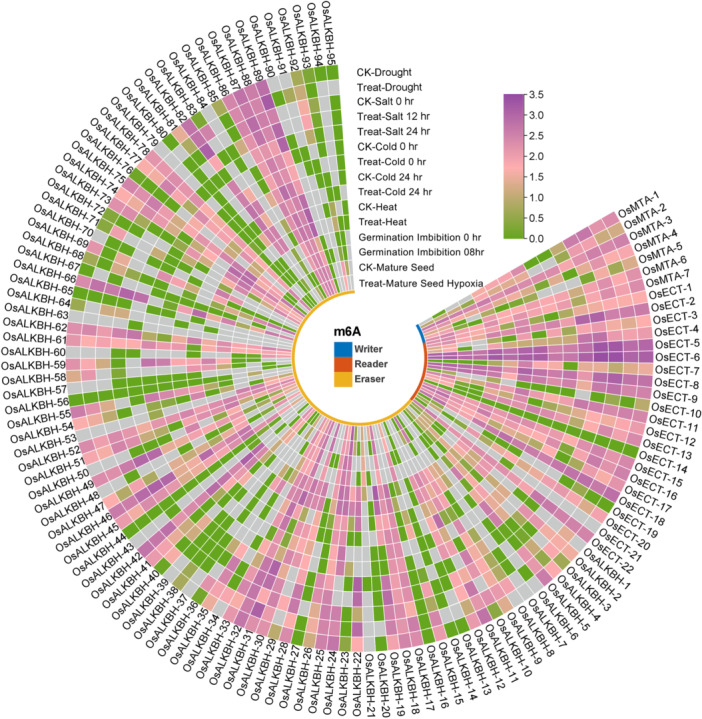
The circular heatmap depicts the stress‐responsive expression profiles of m6A regulatory genes derived from RNA‐seq data. The inner ring colors distinguished the three subgroups of m6A regulators: writer (blue), readers (red), and erasers (yellow). Each column shows the expression levels of individual genes under various stress conditions compared to their control (CK) condition, while the rows correspond to different abiotic stress treatments. The expression values are presented as log2‐normalized data. The color scale denotes relative transcript abundance, with purple indicating high expression and a transition from green to gray representing low or absent expression.

Subsequently, using RNA‐seq analysis, the expression patterns of the Osm6A family were identified in response to various environmental stressors, including drought, salt, cold, heat, germination imbibition, and mature seed hypoxia, as well as their respective control (CK) (Figure 9). The expression profiles of the “writer“ gene group, particularly OsMTA‐1 to OsMTA‐7, showed varied responses to stress conditions, with significant upregulation observed specifically under salt, drought, and heat stress. This consistent activity across multiple stressors suggests that the m6A methylation mechanism is responsive to environmental signals and may contribute to transcriptional regulation associated with stress adaptation. Notably, OsMTA‐1, OsMTA‐3, OsMTA‐6, and OsMTA‐7 exhibited strong expression patterns, with upregulation in most stress conditions tested compared to the CK. In contrast, OsMTA‐2 and OsMTA‐5 appeared either downregulated or showed negligible expression in specific treatments. The “writer” genes were also upregulated during germination and seed hypoxia, indicating their crucial roles in seed activation and responses to oxygen‐deprived conditions. These findings suggest that rice plants enhance the regulation of m6A‐modified RNA molecules in response to stress. Next, the “reader“ genes exhibited a more diverse and specific expression profile. Genes such as OsECT‐5, OsECT‐6, OsECT‐8, OsECT‐9, and OsECT‐18 were significantly upregulated in response to specific stress conditions. Furthermore, OsECT‐5 and OsECT‐9 show significant upregulation under cold, salt, and drought stress, suggesting a role in recognizing m6A‐modified transcripts under these adverse conditions. Notably, OsECT‐2 shows time‐specific up‐regulation during 12 and 24 h of salt (NaCl) treatment. Conversely, the reader genes exhibited lower expression or downregulation under heat stress and hypoxia, reflecting specific functional activity in post‐transcriptional regulation. The expression profiles of the “eraser“ genes were more heterogeneous, with particular genes, such as OsALKBH‐7, OsALKBH‐21, OsALKBH‐43, and OsALKBH‐63, being strongly induced under specific stress conditions, including drought and germination. In contrast, these findings suggest that m6A demethylation may be selectively employed in response to various developmental or environmental challenges. Overall, these results reveal differential regulation of these gene families, underlying the complex and dynamic m6A regulatory network that may support adoptive gene expression programs in rice under specific environmental stresses.

### 3.7 PPIs Network Analysis of Osm6As

**Figure 10:**
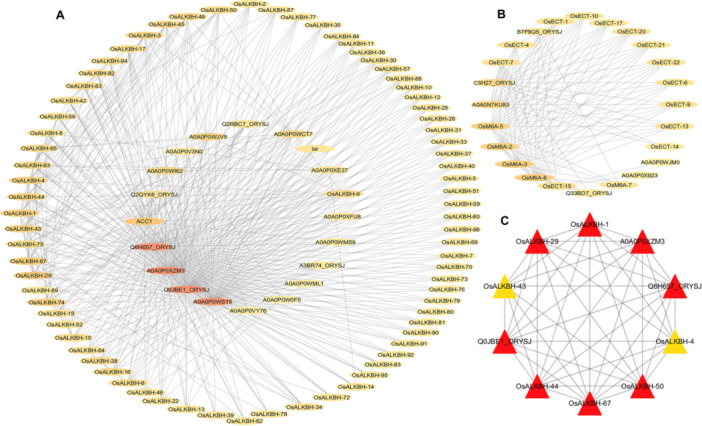
The analysis of protein–protein interaction (PPI) networks involving m6A regulatory genes in rice was illustrated. (A) The interaction network for the eraser group genes was constructed utilizing the STRING database, with a minimum required interaction score set at 0.4, indicative of medium confidence. Additionally, the maximum number of interactors for the first and second shells was limited to 20 and 5, respectively, and the PPI network was visualized using Cytoscape. Visualization, circular nodes represent individual proteins, with the color gradient of the inner circle reflecting the node degree (interaction strength); darker shades indicate a higher number of network connections. (B) The PPI network for m6A was categorized into two subgroups based on the interactions between writers and readers, thereby illustrating the predicted functional associations between these two gene classes and their interaction partners. (C) A highlighted subnetwork shows the top 10 hub genes identified by degree centrality, as assessed by the Cytoscape network analyzer plugin, cytoHubba. The hub nodes are depicted as red or yellow triangles, with red indicating the genes with the highest degree.

PPIs represent essential components of cellular mechanisms and their functional interactions. To obtain a comprehensive understanding of the underlying role and network of Osm6As, the STRING database was utilized. The PPI network generated for all three subgroups comprised 104 out of 124 Osm6As. It is noteworthy that all Osm6As underscore its significance within the context of the three family genes identified for Osm6As (writer, reader, eraser) (Figure 10). Furthermore, in this study, the PPI network demonstrated interactions among several pivotal proteins, including A0A0P0WS15, Q0JBE1_ORYSJ, A0A0P0XZM3, ACC1, and Lar, which are represented in darker colors, indicating higher node degrees as illustrated in the inner circle (Figure 10A). Furthermore, the PPI network of “Writer” and “Reader” proteins forms an integrated group, while the “Eraser” protein occupies a distinct cluster (Figure 10B). This observation provides critical insight into the functional organization of m6A modification. These results indicate a distinct interaction module characterized by the clustering of writer (methyltransferases) and reader m6A‐binding proteins, which broadly coordinates the activities of m6A “Writers” and “Readers” on mRNA. Conversely, the distinct and separate clustering of “Eraser” (demethylase) proteins from the “Writers” and “Readers” groups suggests a significant independent regulatory mechanism for m6A removal. Notably, these identified proteins have consistently demonstrated indispensable roles in responding to environmental stress, as well as in growth and development, including seed development, plant growth, and stress adaptation. Furthermore, this study identified the top 10 hub genes, which form a cohesive module within the Osm6As PPI network and potentially represent the core machinery of m6A regulation (Figure 10C). These findings provide valuable insights into the potential biological functions and molecular mechanisms of Osm6As.

### 3.8 GO and KEGG Enrichment Analysis

**Figure 11:**
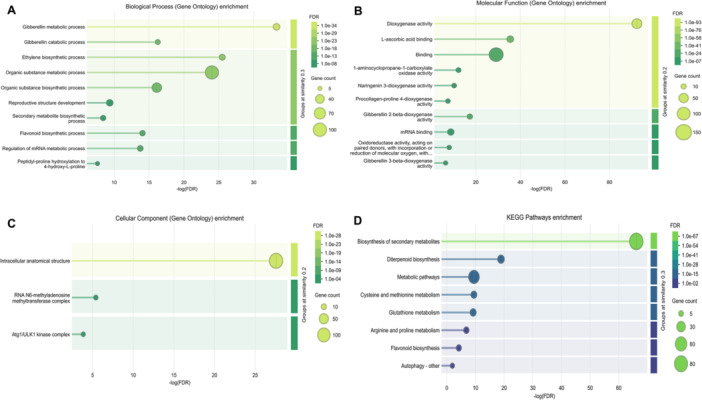
Gene Ontology (GO) and KEGG pathway enrichment analysis were conducted using log‐transferred false discovery (log FDR) counts. The bubble plot represents GO terms categorized (A) Biological Process, (B) Molecular Function, (C) Cellular Components, utilizing the STRING database. The color and size of bubbles correspond to the FDR value and gene counts, respectively. The x‐axis denotes the enrichment score, while the y‐axis lists the distinct biological and functional associations within Osm6A across all subgroups.

To elucidate the functional annotation and enrichment, Go and KEGG pathway analyses were performed. Three GO categories were employed: BP, MF, and CC. The GO analysis identified 49 entries that were significantly enriched across these three GO term categories. Of these, 19 entries were identified in the BP in which the three most predominant terms were “GO:0071704” (organic substance metabolic process), “GO:1901576” (organic substance biosynthetic process), and “GO:0009685” (gibberellin metabolic process) (Figure 11A). The GO term MF identified 15 entries, with the most predominant term including “GO:0005488” (binding), “GO:0051213” (dioxygenase activity), and “GO:1901363: (heterocyclic compound binding) as shown in Figure 11B. Lastly, the GO term CC analysis detected 3 entries, such as “GO:0005622” (intracellular anatomical structure), “GO:0036396” (RNA m6A methyltransferase complex), and “GO:1990316” (Atg1/ULK1 kinase complex) as illustrated in Figure 11C. Furthermore, KEGG pathway enrichment analysis of proteins associated with the rice m6A regulatory network revealed eight key pathways that significantly highlight the diverse functional roles of these genes in stress adaptation, signaling, development, and metabolism (Figure 11D). Notably, these pathways are associated with abiotic and biotic stress, growth and development, singling, m6A regulation, stress response, heat, salt, drought, pathogen resistance, UV‐light defense, and stress recovery senescence. In contrast, these findings provide comprehensive insight into the network associated with Osm6As genes, connecting molecular, cellular, and biological functional enrichment pathways that play a crucial role in crop plants. The extensive involvement of these pathways in developmental regulation and stress adaptation suggests that m6A modification may enhance resilience in response to the adverse effects of climate change in rice.

### 3.9 Subcellular Localization of YTH‐Domain OsECT2

**Figure 12:**
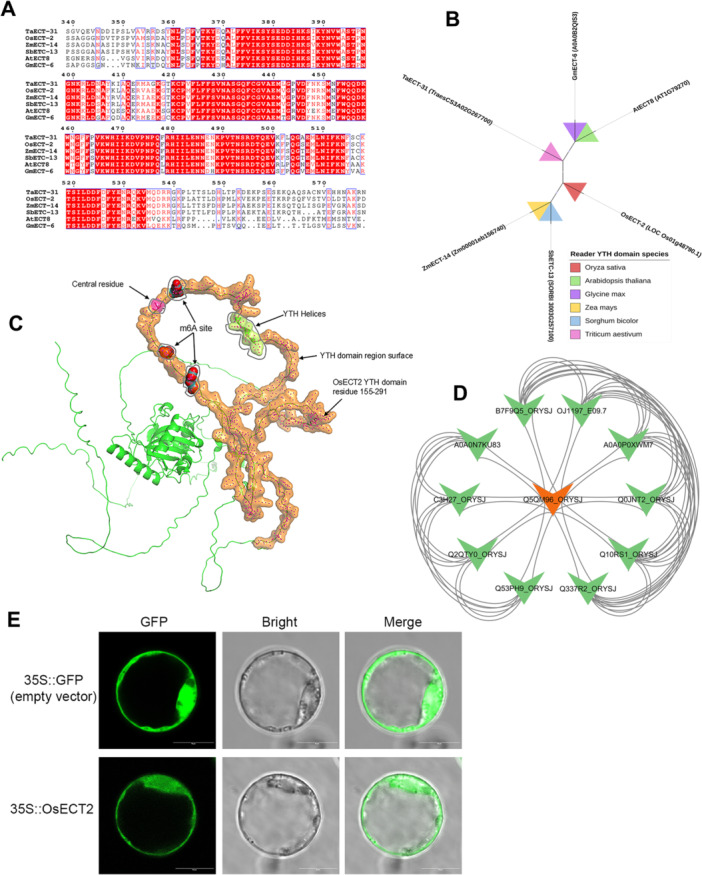
Subcellular localization and structural analysis of OsECT‐2 as a potential m6A reader in Rice. (A) A multiple sequence alignment of the YTH domain across different species. The conserved residues involved in m6A binding are indicated by a red background with 100% identity. (B) Phylogenetic relationship of ECT‐2 orthologs from six plant species. The color‐coded wedges represent distinct species. (C) The predicted 3D structure of OsECT‐2 shows the YTH‐domain (residues 155–291, highlighted in orange surface rendering) forming a classical aromatic cage for m6A recognition. Three α‐helices, part of secondary structure, are shown in green ribbons of the m6A site. The central aromatic residues were predicted in pink, while m6A sites are marked in red color, demonstrating structural compatibility with m6A readers. (D) The STRING‐based interaction network displays predicted interaction of OsECT‐2 (highlighted in orange). (E) Subcellular localization of OsECT2 in rice protoplasts. Green fluorescent protein (GFP) was fused to the C‐terminal of OsECT2 and driven by the CaMV 35S promoters.

To explore the potential function of the m6A modifier, multiple sequence alignments were used to identify homologous residues in the biological sequences. The multiple sequence alignment of OsECT2 with ECT8 homologs in Arabidopsis revealed highly conserved regions within the YTH domain, particularly residues critical for m6A recognition (Figure 12A). The phylogenetic analysis clustered OsECT2 with AtECT8 and other monocot homologs, revealing functional conservation across species. Notably, OsECT‐2 was closely grouped with ZmECT from maize and SbECT from sorghum, both monocot species (Figure 12B). The 3D structure of the YTH domain and m6A‐binding pockets using AlphaFold (Q5QM96) and PyMOL visualization showed well‐defined α‐helices and the m6A binding pocket consistent with its predicted role as an m6A reader (Figure 12C). The PPI network analysis revealed that OsECT‐2 (Q5VQI6_ORYSJ) functions as a central hub interacting with several proteins. These proteins were annotated, and their interactions were mapped, including A0A0P0XWM7 (Zinc finger C2H2‐type) and Q10RS1_ORYSJ (MT‐A70‐like). Notably, Q0JNT2_ORYSJ, identified as an N6‐adenosine‐methyltransferase non‐catalytic subunit like METTL14, was a key interactor, suggesting display identified as a prominent interactor, exhibiting MT‐A70‐like domains characteristic of m6A writers Figure 12D. These results suggest the interaction between OsECT‐2 (readers) and METTL14‐like protein (writers) forms a coordinated epitranscriptomic regulatory system.

Based on the conserved YTH domain and predicted nuclear localization, we constructed a 35S::OsECT2‐GFP vector and transiently expressed it in rice protoplasts. An empty GFP vector (35S::GFP) was utilized as a control. To investigate the biological functions of OsECT2, we analyzed its subcellular localization by amplifying and fusing OsECT2 to the C‐terminus of enhanced green fluorescent protein (GFP), driven by CaMV 35S promoters. The results revealed that OsECT2‐GFP exhibited predominantly nuclear localization, while the control GFP showed diffuse fluorescence throughout both the cytoplasm and nucleus (Figure 12E). In contrast, our findings revealed a strong fluorescence signal specifically enriched in the nucleus. These pronounced nuclear accumulations of OsECT2 provide compelling biological evidence that this m6A reader operates at the nuclear level, where the deposition and processing of methyladenines (m6A) primarily occur, potentially regulating post‐transcriptional gene expression.

### 3.10 Gene Expression Analysis of Osm6As by qRT‐PCR

**Figure 13:**
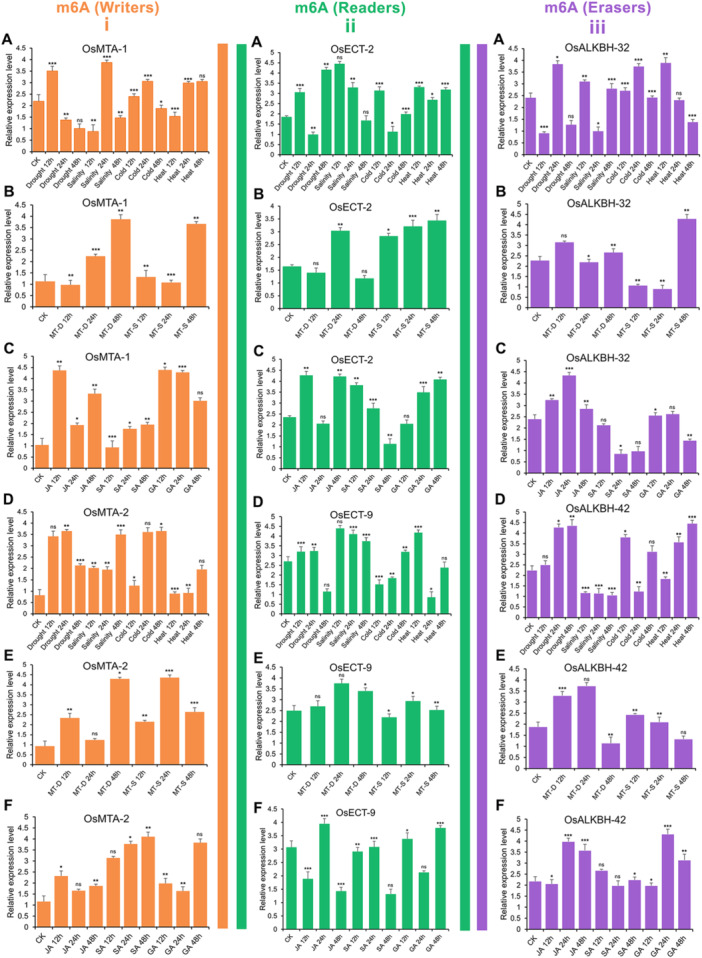
The validation of selected Osm6A regulatory genes in response to abiotic stress and phytohormonal treatments was conducted using quantitative real‐time PCR (qRT‐PCR) analysis. The relative expression levels of genes from three subgroups were categorized into three groups: Writers (i) (A–F), Readers (ii) (A–F), and Erasers (iii) (A–F). Each gene expression profile is presented in a separate panel (A–F). The colors of the bars denote gene groups: Writers (orange), Readers (green), and Erasers (purple).

The m6A pathway, the most prevalent RNA modification, is emerging as a critical epitranscriptomic marker that regulates gene expression and exhibits a capacity for rapid adoption in response to environmental stresses [18]. In order to investigate the gene expression profiles of Osm6As in response to diverse stresses, including hormonal treatments. The expression patterns of six Osm6As genes were significantly regulated in response to abiotic stress. Notably, the OsMTA‐1 gene exhibited a dynamic transcriptional response under different stress conditions, showing a significant increase in expression during drought stress at the 12‐h treatment (*p* < 0.001), while a downregulation was observed at 24‐h treatment compared to control (Figure 13A). In contrast, salt stress resulted in an upregulation of expression at the (24 h) mark at (*p* < 0.001) (Figure 13B). These findings align with those reported by Hu, Cai, Park, Lee, Li [59], which indicated an increase in the expression of the m6A writer subunit following high concentrations of NaCl treatment. Importantly, in this study, two genes from the writer group, OsMTA‐1 and OsMTA‐2, were significantly upregulated at the 150 mM NaCl treatment at both 24 and 48 h (*p* < 0.01) and (*p* < 0.001), respectively, as illustrated in the writer group (Figure 13A,D). Furthermore, to investigate the gene expression profiles of Osm6As in response to exogenous melatonin, our results showed increased expression of OsMTA‐1 observed at (*p* < 0.05) at 24 h under drought and salt stress (Figure 13B). Consequently, hormone treatments elevated the expression level to (24 h) marks *p* < 0.001, while the expression of GA remained unchanged in OsMTA‐1. Additionally, OsMTA‐2 was significantly upregulated under various environmental stresses, including heat and salinity, with *p* < 0.01 and *p* < 0.001 at 24‐h treatment. However, no significant changes were noted under JA and SA treatments.

The Arabidopsis (ECT8), as a member of the m6A “Readers” protein, has demonstrated m6A‐binding capabilities, which correlate with a significant upregulation of gene expression in response to various abiotic stresses, including salt stress [60]. The current study indicates that OsECT‐2 exhibits a substantial response to salt stress after 24 h at (*p* < 0.0001), and a response to JA treatment is also notable at *p* < 0.01, as illustrated in the readers group (Figure 13A,F). Notably, its expression in response to cold and exogenous melatonin with drought stress MT‐D at 12‐ and 48‐h intervals of treatment was found to be non‐significant, suggesting a stress‐specific activation pattern, while in salt stress, MT‐S at 24 h was found to have notable upregulation at *p* < 0.001. Moreover, the OsECT‐9 gene showed a pronounced response to MT‐D after 48 h, with significance at (*p* < 0.05), whereas during the same 12 and 24 h of treatment, the expression was found to be upregulated; nonetheless, no significant observations were detected during 12 and 24 h. In the current study, both genes exhibited significant phase‐specific responses in SA‐treated plants, consistent with previous findings that AtECT1 mediated the SA response in Arabidopsis [47]. These findings underscore distinct transcriptional changes associated with m6A readers. Furthermore, the observed selective expression patterns corroborate prior research indicating significant upregulation of reader genes across various abiotic stresses.

The m6A “Erasers,” which are demethylases, play crucial roles in plant development and responses to environmental stressors, including salt and drought [61]. To elucidate the critical role of m6A “Erasers,” this study examined alterations in gene expression under specific stress conditions induced by abiotic factors, including exogenous melatonin and hormonal treatments. OsALKBH‐32 exhibited a highly significant induction after 24 h of cold stress (*p* < 0.001) and heat stress (*p* < 0.01), as well as under drought and salt stress. Whereas it showed minimal responses to hormonal treatments and early MT‐D exposure. These observations coincide with exciting literature indicating that m6A “Erasers” are activated by extreme temperatures [4]. Furthermore, OsALKBH‐42 was upregulated under drought stress after 48 h (*p* < 0.01), whereas its expression was reduced under salt stress compared with the control; however, significant upregulation was maintained across 12–48 h of treatment (*p* < 0.001). Additionally, this study found significant upregulation of OsALKBH‐42 during MT‐D exposure for 12 h (*p* < 0.001) and under drought stress after 48 h (*p* < 0.01), while no significant response was observed with GA and SA. Collectively, these findings suggest that m6A modifiers, categorized into three distinct families, “Writers” (MT‐A70 family), “Readers” (YTH family), and “Erasers” (ALKBH family), support a regulatory pattern of gene expression that is differentially activated depending on the type and duration of stress. This study underscores their potential role as molecular targets for enhancing stress resilience in rice to adverse environmental stressors, including plant growth and development.

## 4 Discussion

Unpredictable adverse environmental conditions, encompassing abiotic stresses such as drought, salinity, ultraviolet light, extreme temperatures, elevated CO_2_, and heavy metals, significantly hinder agricultural productivity and disrupt nutritional balance. These challenges pose a substantial threat to food security [62, 63, 64, 65, 66, 67]. Among these stresses, salinity is particularly detrimental, as it adversely affects crop productivity. Specifically, high salinity represents a significant obstacle to plant growth and development. Therefore, identifying new genetic mechanisms for high salinity tolerance is an effective strategy for breeding salt‐tolerant genotypes [65]. Furthermore, a comprehensive understanding of the mechanisms underlying plant responses to melatonin, a critical hormone involved in biological functions, is essential for developing strategies to enhance stress tolerance and promote agricultural sustainability under challenging environmental conditions. The discovery of chemical modifications in RNA that constitute the “epitranscriptomic” plays an essential role in regulating gene expression and various molecular and cellular processes, including plant adaptation to stress resilience and signaling [4]. Subsequently, the discovery of the m6A pathway and its modification in eukaryotic cells has provided new insights into mRNA epigenetics, elucidating these modifications as additional tiers in epigenetic regulation [68].

The identification of gene families associated with m6A modifiers has been extensively characterized across various plant species, including S*olanum lycopersicum* [69], *litchi* [70], poplar [71], and *Aegilops tauschii* [72]. Comparative phylogenetic analysis of m6A gene families across monocots and dicots among six model crop species, *Oryza sativa, Arabidopsis thaliana*, *Triticum aestivum*, *Zea mays, Sorghum bicolor*, and *Glycine max*, revealed functional and divergent evolutionary relationships within these model crops, indicating a conserved evolution of m6A‐related gene families. Despite the evolutionary distance among these species, the phylogenetic analysis identified several clusters that appeared across monocots and dicots, suggesting a broad regulatory role for the m6A modifier. These findings provide valuable insight for gene functional studies, particularly in understanding complex genomes. In contrast, understanding these evolutionary trends may enhance breeding strategies for future RNA modifications. Gene duplication is widely recognized as a crucial mechanism for generating genetic variation, serving as the foundational substrate for the emergence of new genes and the development of novel functions [73, 74]. A gene family consists of two or more copies of a gene that arise through gene duplication [75]. The three primary modes of gene duplication are SD, tandem duplication, and transposition events. In a tandem gene duplication event, two or more identical or similar copies of a gene are situated adjacent to one another. Conversely, segmental gene duplication of larger genomic regions typically results from chromosomal rearrangements [76]. In plants, segmental and tandem duplications are crucial mechanisms that expand gene families. It has been predicted that WGD or SD events will lead to the expansion of the Osm6A‐related gene families. Correlated gene arrangements among taxa are critical for inferring the shared ancestry of genes [57]. Homologous genes, which originate from a common ancestor, are preserved on corresponding chromosomes (synteny) and maintain a consistent order of arrangements (collinearity) throughout the course of evolution [77, 78].

Synteny and collinearity analyses of the rice genome compared with those of *Arabidopsis thaliana*, *Sorghum bicolor*, and *Glycine max* suggest that the rice genome has undergone a triplication event. Furthermore, tandem duplication has significantly contributed to the expansion of the Osm6A‐related gene family. These events have led to a rapid proliferation of the m6A gene family in rice, resulting in a notably higher number of identified Osm6A genes compared to other plant species, including *Aegilops tauschii* [72], *Solanum Lycopersicom* [69], and *litchi* [70, 79]. As well as other crops such as *Zea mays* and *Brassica napus*. Plants have consistently encountered various environmental stresses throughout their evolutionary period. In response to these adverse conditions, plants have developed sophisticated strategies, with TFs playing a crucial role. TFs regulate the mRNA abundance of numerous downstream target genes by interacting with cis‐acting elements in the promoters of these genes. Several TF gene families and their associated cis‐elements are key players in responses to abiotic and biotic stresses [80]. In this study, the analysis of 2000 bp upstream regions of promoters revealed consistent detection of key cis‐acting elements, indicating that these genes are subject to complex multi‐layered transcription regulation in response to environmental stimuli. Notably, the utmost TATA‐box was detected in all Osm6A “writer” genes, as illustrated in Figure 4. These findings reflect the essential role of these elements for transcription initiation; our results were consistent with [81]. In addition, the cis‐acting elements related to TFs and stress‐responsive, such as MYB recognition involved in drought, salt, and pathogen defense mechanisms, MYC response to ABA and JA signaling involved in abiotic stress, G‐Box, ABRE, and P‐Box are responsible for phytohormonal regulation, such as ABA and GA, including drought, salt, light growth, and development.

The “Reader” group of m6A genes, a distinct cis‐acting element, was identified, indicating that these genes are involved in broad environmental stress responses facilitated by TFs and hormone signaling networks. Notably, motifs such as STRE, ABRE, G‐box, MYB, MYC, and TCA elements were identified, all of which are well‐established regulators of abiotic stress responses, particularly salt, drought, and temperature variations, and are closely associated with ABA, JA, and SA hormonal pathways (Figure 5). Among these readers, OsECT2 was found to be particularly enriched with ABA‐responsive motifs (ABRE, ABREa, ABRE4) and stress‐inducible motifs such as (STRE, TC‐rich, MYB, and MYC), suggesting a high degree of transcriptional landscape corresponds with our qRT‐PCR and transcriptome analyses, which demonstrated that OsECT2 was significantly induced under salt stress, thereby reinforcing its role as a key m6A reader that coordinates epitranscriptomic regulation with hormonal and stress signaling. Next, the Osm6A “Eraser” demethylation group exhibits a significant prevalence of the WUN‐motif, W‐box, ERE CGTCA, and TGACG elements. These motifs are mainly associated with biotic stress, including wound response and mechanical damage, and the W‐box being bound by WRKY TFs, which play a crucial role in abiotic and biotic stress. In contrast, these motifs are associated particularly with biotic stress, including secondary metabolism, ETH, JA, and development regulation, illustrated in (Supporting Information: Figures S4 and S5). This study reveals a distinct underlying mechanism through which epitranscriptomic regulation may be integrated with defense pathways and hormonal signaling, particularly under abiotic stress conditions. Furthermore, all three families of Osm6A (writer, reader, eraser) exhibit a comprehensive regulatory mechanism. Overall, our findings indicate that OsECT2 integrates its mRNA methylation function with promoter architecture that enables responsiveness to abiotic stress, particularly salt stress. This dual layer of regulation involving cis‐acting elements and post‐transcriptional m6A readers positions OsECT2 as a key molecular hub in rice's response to salt stress, providing a new target for developing climate‐resilient crops.

A total of 124 Osm6A‐related genes were mapped to 11 chromosomes, with 7 genes from “Writer” located on chromosomes 1, 3, and 10. In addition, 22 Osm6As “reader” genes were identified on Chr1, 3, 4, 5, 6, 7, and 8, while 95 “eraser” genes were found distributed across all 11 chromosomes. Notably, no genes were identified on chromosome 12. The majority of gene families associated with the m6A pathway appear to be evenly distributed across the 11 chromosomes. These findings suggest that the majority of duplications of the Osm6As genes likely occurred before the divergence of *Arabidopsis thaliana*, *Sorghum bicolor*, and *Glycine max*. Additionally, gene deletions and chromosomal rearrangements occurred throughout the evolution of the rice genome. Collinearity analysis is a crucial tool in comparative genomics, enabling researchers to investigate molecular evolutionary events across species and to provide valuable insights into functional predictions. The extent of covariance segments indicates temporal divergence among species; species that diverged more recently exhibit reduced variation and retain more ancestral features [77]. Conversely, species with more extended divergence periods tend to share fewer traits due to the accumulation of genetic variation over time [82, 83]. Notably, the collinearity relationships among rice genomes and those of *Arabidopsis thaliana* and *Glycine max* showed divergence, suggesting that this may be due to chromosomal rearrangements or diversions post‐monocot‐dicot split, consistent [84]. Gene duplication plays a vital role in the evolution of organisms, significantly increasing their complexity over time. Segmental or WGD is a fundamental driver of gene family evolution in response to diverse environmental stressors. For example, the rice genome and sorghum share evidence of ancient WGD events, which suggests retained duplication aligned with our finding [85]. In crop plants, WGD events often retain genes involved in transcriptional regulation and stress‐responsive genes, while housekeeping genes tend to be reduced [86]. Our findings suggest that comparative genome duplication analysis often retains the notable differences among duplicate gene pairs in the Osm6As [48, 87].

Previous studies have underscored the involvement of m6A modifications in numerous cellular and biological functions during plant responses to abiotic and biotic stress, including plant growth development and stress response. The consequences of these modifications have been reported since their initial identification in eukaryotes [88], up to the present. Specifically, in abiotic stress, particularly drought stress [89]. Salt stress [59, 90, 91]. Heat stress [92]. Low temperature stress [93]. Cadmium stress [94]. In biotic stress, such as rice stripe virus or rice black‐stripe dwarf virus infection [95]. Sea buckthorn (*Hippophae rhamnoides*) [96]. *Cucumber green mottle mosaic virus* (CGMMV) [97]. Peanut (*Arachis hypogea* L.) resistance to bacterial wilt (BW) [98]. These findings suggest that the m6A‐regulatory mechanism has diverse biological functions in plants, particularly under abiotic stress conditions. It has been demonstrated that ECT8 expression is significantly upregulated under salt stress [60]. Consistent with this, our findings corroborate and expand upon earlier reports: AtECT8, along with its orthologs in rice OsECT‐2, OsECT‐3, and OsECT‐4, which cluster within the same phylogenetic clade, exhibit a high expression pattern in response to salt stress [17]. This conservation of stress responsiveness across species underscores the potential functional importance of YTH‐domain‐containing m6A readers in salt‐stress adaptation.

Previous research has revealed that Arabidopsis encodes 11 ECT proteins, of which only a few members, such as ECT2, ECT3, and ECT8, have been functionally characterized [99]. Among these, ECT8 has been shown to enhance salt tolerance by stabilizing positive stress regulators via interactions with m6A‐modified mRNAs. Conversely, loss‐of‐function mutants of ect8 exhibit hypersensitivity to salinity [17]. Additionally, Arabidopsis ECT1 promotes plant growth and development via GA signaling, with its mutants displaying reduced responsiveness to GA_3_ during seed germination [100]. In the present study, we conducted a focused functional characterization and expression of OsECT2, a homolog and cluster within the same phylogenetic clade as AtECT8. Our findings indicate that OsECT2 is significantly up‐regulated by melatonin under salt stress, and its YTH‐domain architecture strongly suggests a conserved function as an m6A reader that mediates post‐transcriptional regulation during abiotic stress. Notably, OsECT2 interacts with predicted METTL14‐like (Q10RS1_ORYSJ) writer proteins and zinc‐finger RNA‐binding factors (A0A0P0XWM7), positioning it as a central component in the epitranscriptomic regulatory network. This implies that OsECT2 coordinates m6A methylation and the recognition of target mRNAs, facilitating the dynamic regulation of mRNA metabolism, including stability and translation at the macromolecular level, which is crucial for stress adaptation mechanisms [101, 102].

It is noteworthy that OsECT2, a homolog of Arabidopsis AtECT8, has previously been reported to regulate and inhibit m6A‐dependent processes. The present study thereby revealed that the nuclear localization of OsECT2‐GFP provides evidence for its predicted subcellular distribution and its role as an ECT‐type YTH domain protein in rice, an m6A reader involved in mRNA recognition and post‐transcriptional regulation. Our discovery aligns with previous reports that the Arabidopsis C‐terminal region (ECT8), an m6A reader, is upregulated in ABA, SA, salt, and osmotic‐stress responses [47, 103]. Furthermore, Arabidopsis ECT1 promotes seed germination via GA signaling [100]. The ect8 mutant, before losing its function, exhibited increased salt sensitivity in Arabidopsis [17]. In addition, the OsYTH gene in rice was knocked out, and the mutants showed more sensitivity to abiotic stress [104]. These reports demonstrate that ECT, as a m6A reader protein, directly binds to m6A to enhance its stability in Arabidopsis. The RT‐qPCR validation of six representative Osm6A genes reinforces their diverse regulatory roles under various abiotic stress and hormonal conditions, including exogenous melatonin. Among these, the “writers” genes OsMTA‐1 and OsMTA‐2 exhibited significant upregulation under distinct stress conditions, indicating a strong role in stress‐associated m6A deposition. The “reader” genes OsECT‐2 and OsECT‐9 demonstrated robust responses to salinity stress (NaCl), MT‐D, MT‐S, and JA, linking m6A‐mediated regulation to hormonal signaling, including melatonin stress adaptation. Subsequently, the “eraser” genes OsALKBH‐32 and OsALKBH‐42 showed increased expression under extreme temperatures and in response to melatonin, suggesting their involvement in maintaining methylation homeostasis during stress responses [105].

Collectively, underscore the biological significance of OsECT2 within the m6A regulatory pathway. The PPI network positions OsECT2 in close association with METTL14, a key m6A writer, suggesting that OsECT2 likely interacts with a coordinated m6A regulatory module. Furthermore, the nuclear localization of the OsECT2–GFP fusion, consistent with our in‐silico prediction and PPI network analysis, provides compelling evidence that OsECT2 functions at the site where m6A recognition and transcript processing occur. Additionally, RNA‐seq and qRT‐PCR analysis demonstrates a significant and time‐dependent upregulation of selected genes, including OsECT2, in response to salt stress following the application of exogenous melatonin. This inducible expression implies that melatonin may enhance stress tolerance, in part through post‐transcriptional m6A‐mediated regulation. Moreover, together, these converging lines of evidence predicted domain features, nuclear localization, expression patterns, and network connectivity, demonstrating that OsECT2 acts as a melatonin‐responsive m6A reader whose activity may modulate transcript fate under salt stress. Future studies that incorporate physiological markers, such as ROS scavenging, osmolyte accumulation, and ion homeostasis, will further elucidate the regulatory insight into the identified m6A‐modifiers. Moreover, the regulatory mechanisms by which these modifiers respond to abiotic stress, particularly salt stress, by activating exogenous melatonin, reveal a new target for engineering stress‐resistant crops in the context of climate change.

## 5 Conclusion

This study is the first to comprehensively identify the role of the m6A modifier in epitranscriptomics, demonstrating how its expression suppresses external melatonin and enhances salt tolerance in *Oryza sativa*. This analysis encompasses the roles of writers, readers, and erasers. We identified 124 m6A‐associated genes, with the majority of their encoded proteins localized to the nucleus, suggesting their potential involvement in mRNA modification. Among these, OsECT2, a YTH‐domain m6A reader, has been identified as a crucial regulator of salt stress adaptation. Our findings demonstrated that OsECT2 binds to m6A‐modified mRNAs and interacts with METTL14‐like writer proteins and RNA‐binding factors, highlighting its significant role in post‐transcriptional regulation during abiotic and hormonal treatment, including melatonin. Phylogenetic and comparative analyses with both monocot and dicot species confirmed the evolutionary conservation and functional diversification of genes associated with the m6A machinery. Importantly, qRT‐PCR analysis identified potential molecular targets for enhancing abiotic stress tolerance. Overall, this study provides a comprehensive framework for understanding m6A‐mediated regulation in rice and offers strategic insights for future functional validation and molecular breeding programs for improving climate resilience and crop productivity.

## Author Contributions

**Hira Khanzada:** conceptualization, data curation, writing – original draft, methodology. **Ghulam Mustafa Wassan:** project administration, formal analysis, software, visualization, writing – review and editing. **Ping Wang:** methodology. **Saba Khanzada:** methodology. **Xiaoning Wang:** resources, funding acquisition, and supervision. **Zhihui Xia:** validation, resources, funding acquisition, and supervision.

## Conflicts of Interest

The authors declare no conflicts of interest.

## Supporting information


**Supplementary Figure S1:** Illustrates the motif distributions and gene structures of the Osm6A erasers (2OG‐fell Oxy super family) commonly known as ALKBH.

**Supplementary Figure S2:** The analysis of cis‐regulatory elements within the 2000 bp upstream region of the transcription start site of the Osm6A writer genes.

**Supplementary Figure S3:** The analysis of cis‐regulatory elements within the 2000 bp upstream region of the transcription start site of the Osm6A reader genes.

**Supplementary Figure S4:** The analysis of cis‐regulatory elements within the 2000 bp upstream region of the transcription start site of the Osm6A eraser genes.

**Supplementary Figure S5:** The heatmap analysis presents the quantitative identification of motifs within the cis‐acting elements of each gene group.

**Supplementary Table S1:** RNA‐seq datasets used for tissue expression analysis of Osm6A genes.

**Supplementary Table S2:**. RNA‐seq datasets used for stress‐condition expression analysis of Osm6A genes.

**Supplementary Table S3:** List of primer sequences used for qRT‐PCR.

**Supplementary Table S4:** Cis‐acting elements analysis of Osm6A (writers) promoter sequences.

**Supplementary Table S5:** Cis‐acting elements analysis of Osm6A (readers) promoter sequences.

**Supplementary Table S6:** Cis‐acting elements analysis of Osm6A (erasers) promoter sequences.

## Data Availability

The data that support the findings of this study are available from the corresponding author upon reasonable request.
