# Supplementary figures and images for: Comprehensive Genome‐Wide Identification and Expression Analysis of the N6‐Methyladenosine (m6A) Regulatory Network Influences Rapid Stress Adaptation With Exogenous Melatonin in Rice
Source: J Pineal Res. 2026 Jan 13;78(1):e70109. doi: 10.1111/jpi.70109 (PMC12796873; doi:10.1111/jpi.70109)

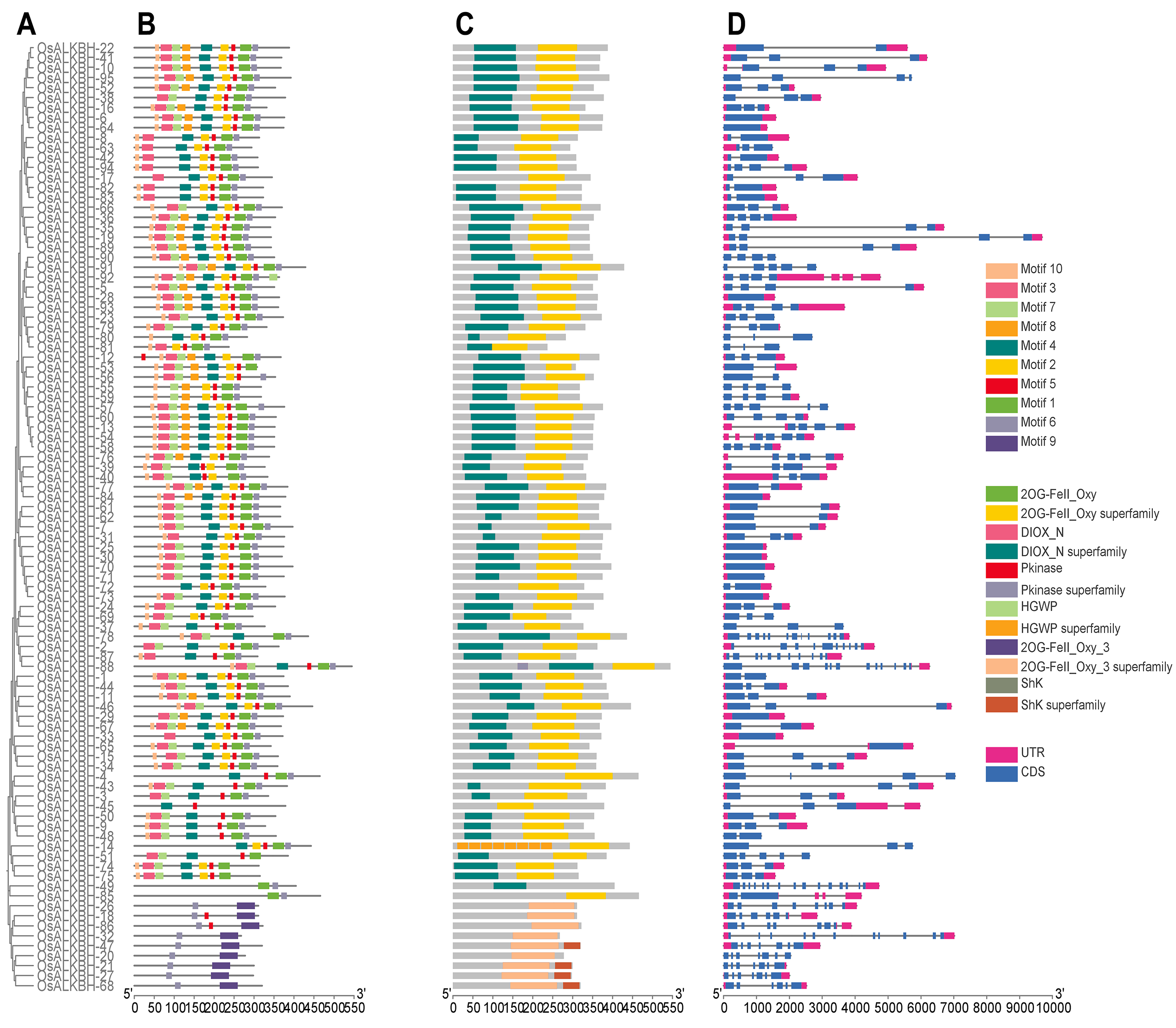

Supplement: Supplementary file 1 — Supplementary Figure S1: Illustrates the motif distributions and gene structures of the Osm6A erasers (2OG‐fell Oxy super family) commonly known as ALKBH. [file JPI-78-e70109-s001.tif]

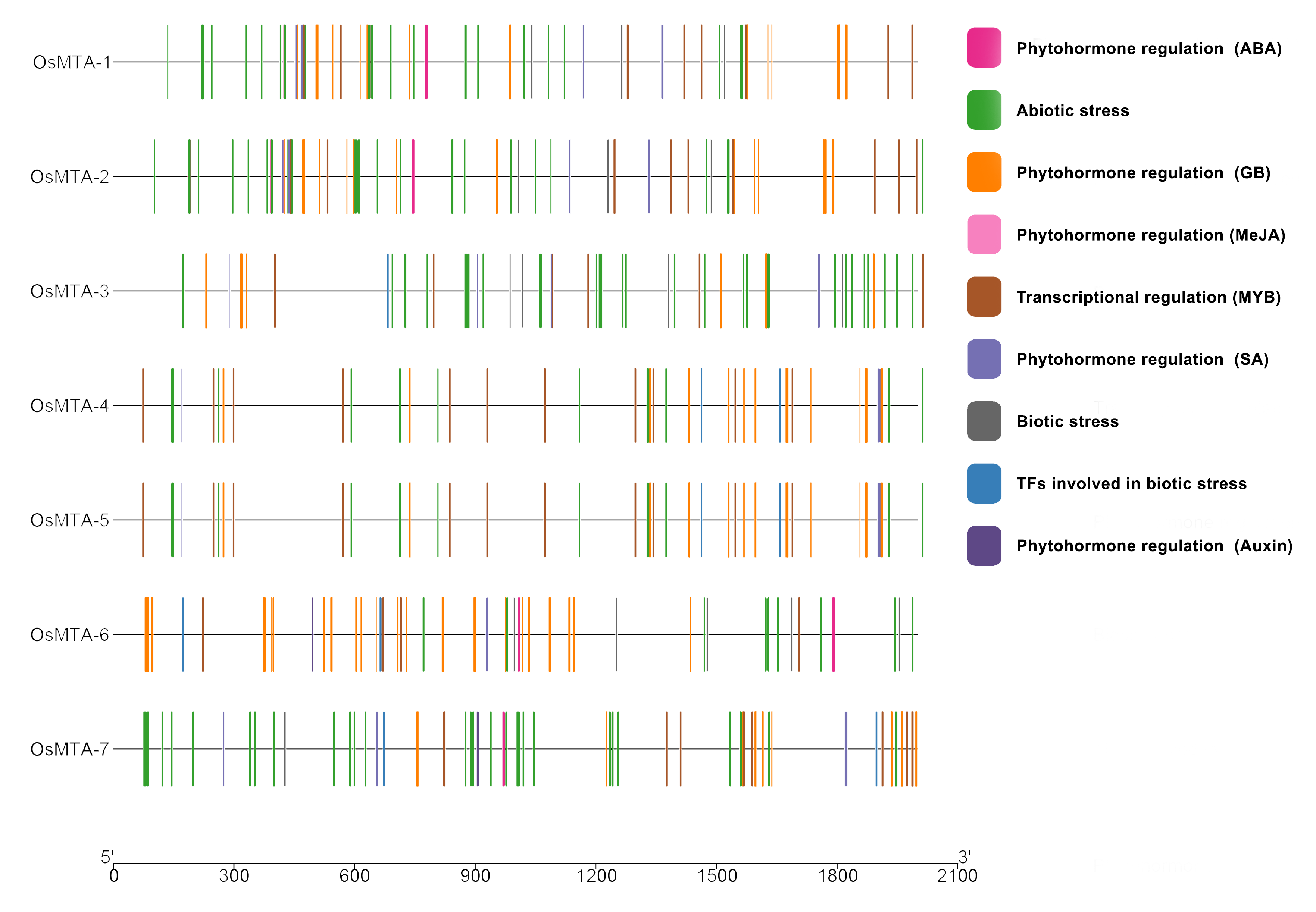

Supplement: Supplementary file 2 — Supplementary Figure S2: The analysis of cis‐regulatory elements within the 2000 bp upstream region of the transcription start site of the Osm6A writer genes. [file JPI-78-e70109-s005.tif]

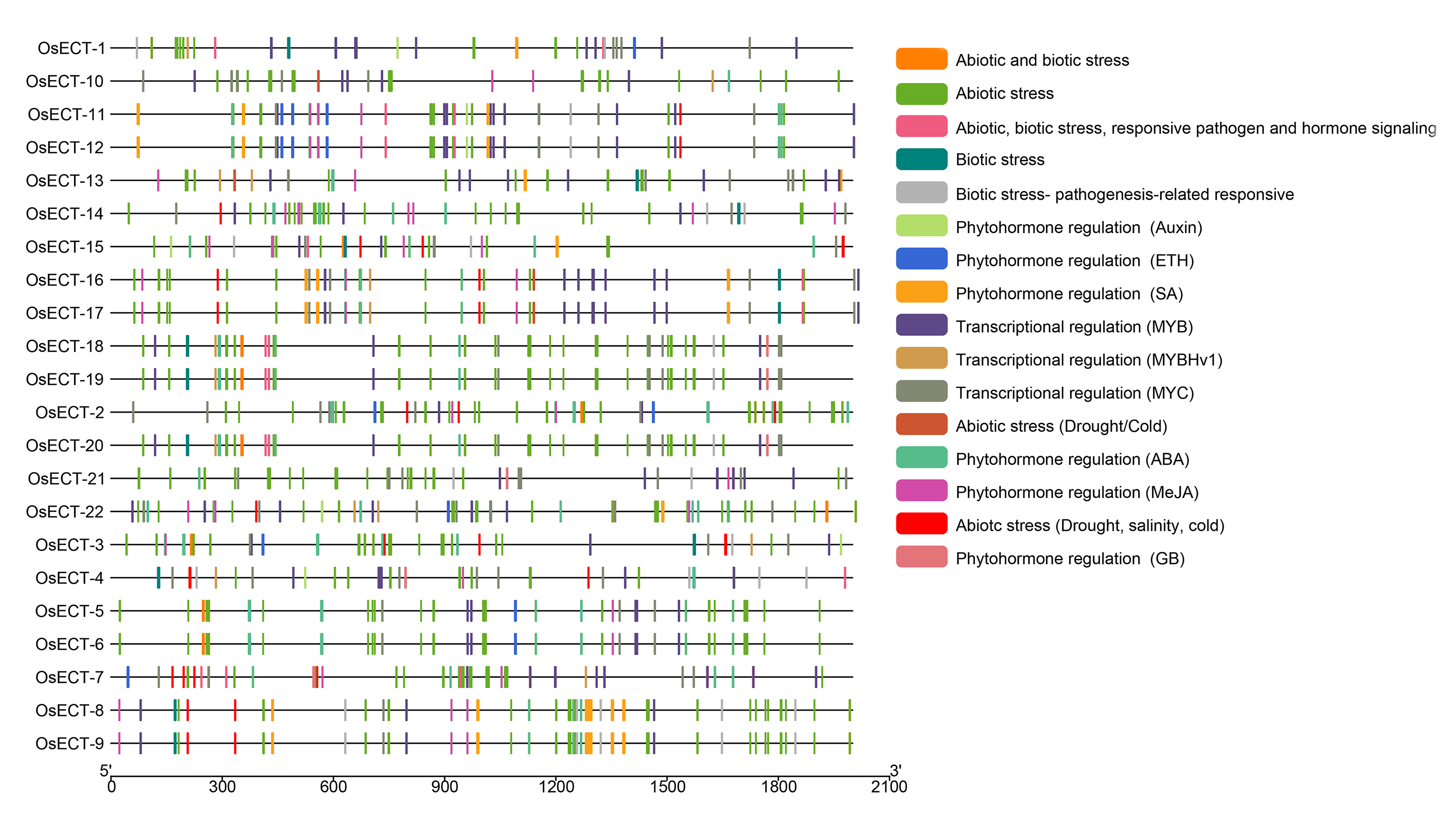

Supplement: Supplementary file 3 — Supplementary Figure S3: The analysis of cis‐regulatory elements within the 2000 bp upstream region of the transcription start site of the Osm6A reader genes. [file JPI-78-e70109-s008.tif]

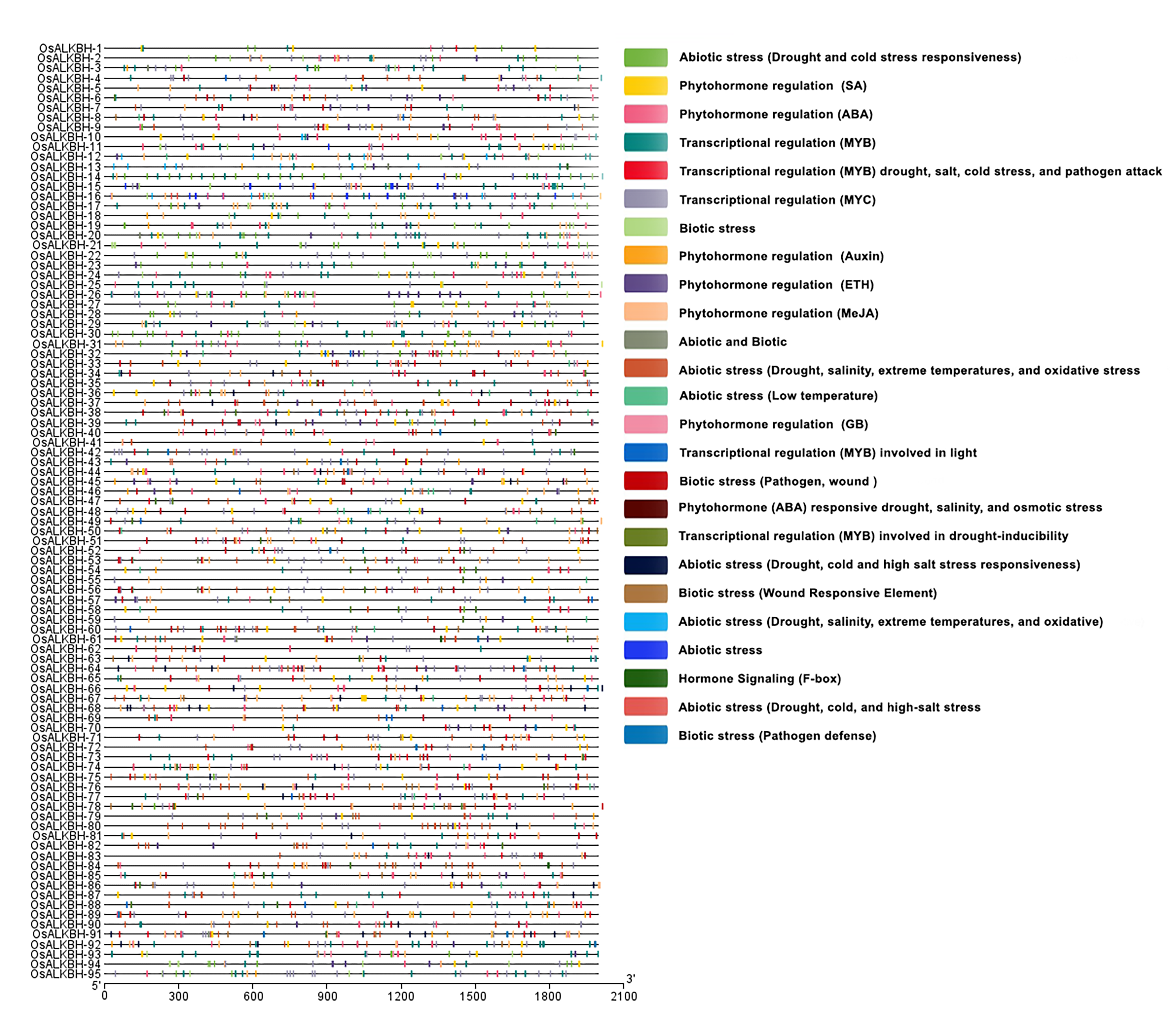

Supplement: Supplementary file 4 — Supplementary Figure S4: The analysis of cis‐regulatory elements within the 2000 bp upstream region of the transcription start site of the Osm6A eraser genes. [file JPI-78-e70109-s006.tif]

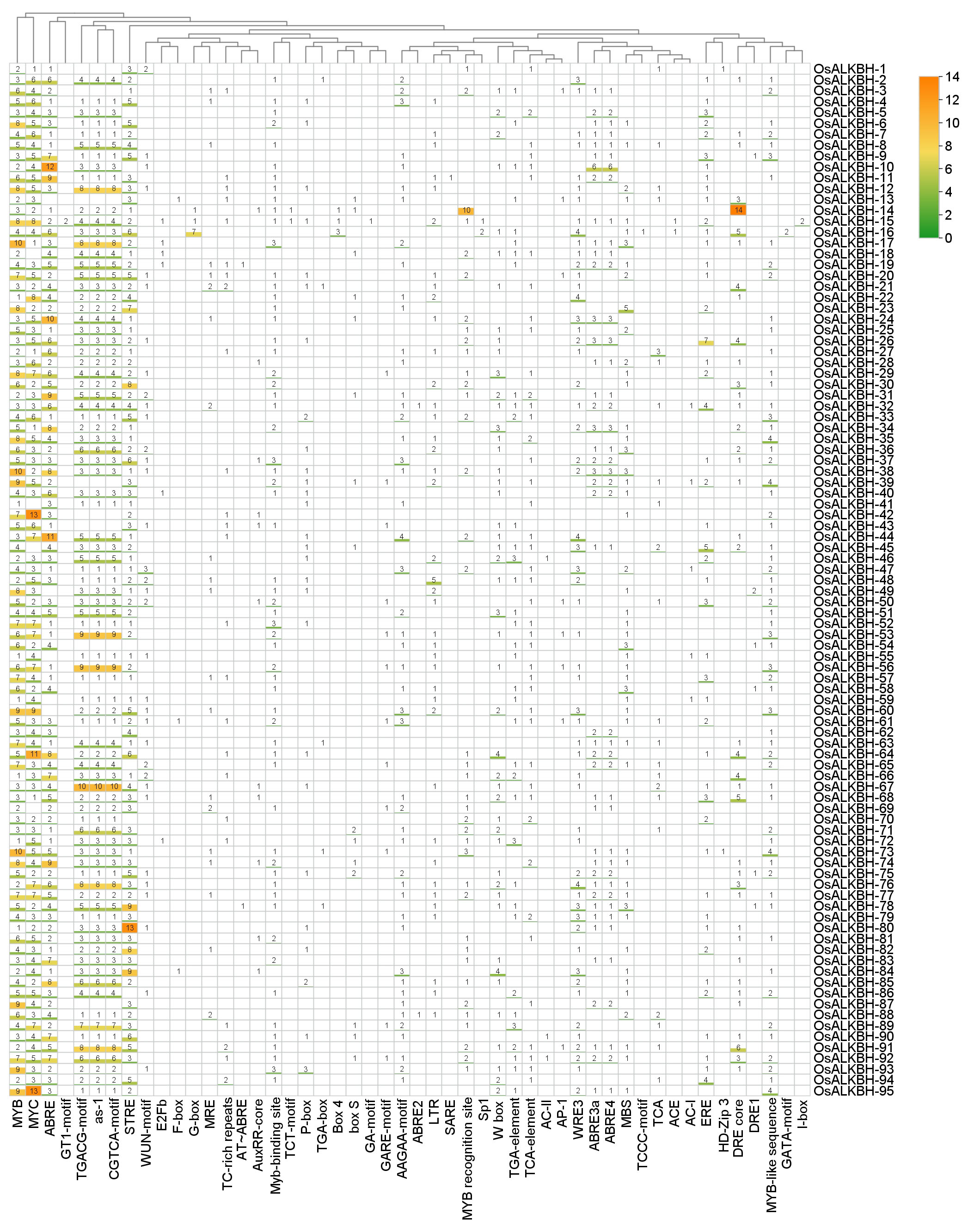

Supplement: Supplementary file 5 — Supplementary Figure S5: The heatmap analysis presents the quantitative identification of motifs within the cis‐acting elements of each gene group. [file JPI-78-e70109-s010.tif]
